# HIV-related data among key populations to inform evidence-based responses: protocol of a systematic review

**DOI:** 10.1186/s13643-018-0894-3

**Published:** 2018-12-03

**Authors:** Amrita Rao, Sheree Schwartz, Keith Sabin, Tisha Wheeler, Jinkou Zhao, James Hargreaves, Stefan Baral, on behalf of the Global.HIV Research Group

**Affiliations:** 1 Department of Epidemiology, Johns Hopkins Bloomberg School of Public Health, 615 N. Wolfe St, Baltimore, MD 21205 USA; 2 Strategic Information and Evaluation, Joint United Nations Programme on HIV/AIDS, Geneva, Switzerland; 3 Office of HIV/AIDS, United States Agency for International Development, Washington, DC USA; 4 Technical Advice and Partnerships Department, The Global Fund to fight AIDS, Tuberculosis, and Malaria, Geneva, Switzerland; 5 Department of Social and Environmental Health, London School of Hygiene and Tropical Medicine, London, UK

**Keywords:** Key populations, Female sex workers, Men who have sex with men, People who use drugs, Incarcerated populations, Treatment cascade, Population size estimation, Systematic review

## Abstract

**Background:**

Key populations who bear a disproportionate burden of HIV, including female sex workers, men who have sex with men, people who use drugs, transgender people, and incarcerated populations, have been understudied, especially in the context of broadly generalized HIV epidemics. Program and investment planning documents often do not take into account the data that do exist. Prior systematic reviews have been comprehensive, but lack sustainability and relevance over time. This review aims to synthesize all available data for key populations and present the data through an accessible, updatable user-friendly graphic interface. The outputs of this systematic review will serve as a resource for decision-makers, providing government stakeholders and donors with the tools to make evidence-based decisions for national planning.

**Methods:**

We will conduct a systematic review of data published or made available between January 1, 2006, and January 1, 2019, that captures the burden of HIV, both prevalence and incidence estimates, HIV prevention and treatment cascades, key population size estimates, experienced violence, consistent condom use, and engagement with healthcare systems for female sex workers, men who have sex with men, people who use drugs, transgender people, and incarcerated populations. A team of reviewers will use Covidence to conduct two independent reviews of both title/abstract and full text for each article. REDCap will be used for data abstraction and storage.

**Discussion:**

Findings from this systematic review and the development of the enhanced graphical interface to display data, along with ongoing efforts to build capacity among key stakeholders to better use and interpret available data, will help ensure that available epidemiologic data related to key populations can be appropriately used to guide large-scale HIV funding and programmatic responses.

**Systematic review registration:**

PROPSERO CRD42016047259.

**Electronic supplementary material:**

The online version of this article (10.1186/s13643-018-0894-3) contains supplementary material, which is available to authorized users.

## Background

As part of efforts to curtail the HIV pandemic and achieve an AIDS-free generation, international directives, including The Global Fund Strategy 2017–2022 and PEPFAR 3.0, have highlighted the need for quality data to inform an effective HIV response [1, 2]. Understanding the scope, distribution, and determinants of country-level HIV epidemics, along with characterizing the effectiveness and ultimate impact of current programs, is needed to improve existing prevention and treatment service delivery systems [1, 2]. For key populations who bear a disproportionate burden of HIV, including female sex workers, men who have sex with men, people who use drugs, transgender people, and incarcerated populations, defining this need has been particularly difficult [3]. Due to stigma, discrimination, and often criminalization, key populations are marginalized and hidden, resulting in greater risks of HIV acquisition and transmission [4–7]. However, these same determinants challenge characterizing the HIV epidemic within these populations and the potential impact on HIV epidemics among all reproductive aged adults. The needs of key populations are often understudied, particularly in high HIV burden settings, where less attention is often given to key populations based on assumptions of limited public health relevance [8–10]. In these generalized epidemic settings, there are often limited data available for female sex workers, gay men and other men who have sex with men, and people who use drugs, and even less data available for other key population groups including transgender women. Traditional approaches, including the static Modes of Transmission (MOT) model, have been used to predict the annual portion of new HIV infections acquired across subgroups. These traditional approaches to estimating the importance of the unmet treatment and prevention needs of key populations do not capture the longer chains of secondary indirect transmissions due to high-risk behaviors and are based solely on static HIV prevalence and assumed population size [11]. These approaches rarely account for underreporting of HIV-associated risks and also tend to treat key populations as isolated groups thereby ignoring the full chains of potential onward HIV transmission [11]. Recent dynamic transmission modeling suggests the importance of addressing the HIV prevention and treatment needs among key populations in all HIV epidemic settings [9, 11–13].

Where data are available for key populations, they are often underutilized in both official policy and program documents used by donors and policymakers [9, 14, 15], possibly due to the lack of political will, the lack of access to relevant data, or the lack of knowledge around the specific issues facing key populations. In an examination of the utilization of key population size estimates in Global Fund and PEPFAR funding proposals and Country Operational Plans (COPs) [14], of the 71 population size estimate studies published between 2009 and 2016 only two were mentioned in Global Fund-related concept notes, 12 in PEPFAR COPs, and seven in national Ministry of Health documents [14]. Despite significant investments to conduct epidemiologic studies of key populations by these donors and an emphasis on the need for high-quality data to inform a response, there exists inadequate evidence of uptake of these data to guide the HIV response.

Overall, there are gaps both in the availability of quality data and utilization of the data of key populations disproportionately affected by HIV. In response to these gaps, the proposed study aims to complete and make available online, a systematic, living, and comprehensive review of all available data for key populations that can be used to inform an evidence-based and human rights affirming HIV response. Data will be reviewed, synthesized, and presented in the form of an enhanced graphical interface. The site will present data in a clear, user-friendly manner to facilitate its use by relevant stakeholders, such as donor governments and international bodies, local government officials, members of key population community leadership as well as affected members of the community, HIV researchers, academics, implementing partners, and the media.

## Methods/design

We will conduct a systematic review of data published or made available between January 1, 2006, and January 1, 2019, that captures the burden and risk of HIV, both prevalence and incidence estimates, prevention indicators and treatment cascades, population size estimates, experienced violence, and engagement with healthcare systems. This protocol is registered in the PROPSERO database (CRD42016047259; 28 September 2016) and is in accordance with the guidelines specified in the Preferred Reporting Items for Systematic Reviews and Meta-analyses statement for protocols [16].

### Objective

- To complete a global systematic review of all available data characterizing the burden of HIV and the HIV treatment cascade among key populations (female sex workers, men who have sex with men, people who use drugs, transgender people, and incarcerated populations) from 2006 to 2019.
- To conduct quality assessments for a subset of all data sources on key populations from 30 priority countries selected based on current HIV programming priorities, on prevalence, incidence, treatment cascades, and population size estimates using a quality assessment tool developed for assessing key populations data.

### Primary outcomes

1. Burden of HIV among key populations as characterized by prevalence and incidence of HIV from 2006 to 2019.
2. HIV treatment cascade among key populations from 2006 to 2019.

Secondary outcomes include prevention indicators (Engaged in HIV testing, knowledge of HIV prevention, condom and PrEP availability, consistent condom use), population size estimates (including specific subnational organizational units to which they apply), experienced violence (physical, sexual, intimate partner), and engagement with healthcare systems for key populations [female sex workers, men who have sex with men, people who use drugs, transgender people, and incarcerated populations], and to make these data updatable and available in the form of an enhanced graphical interface.

### Information sources and search strategies

In partnership with information management specialists at Johns Hopkins University, we will search the following databases: PubMed©, EMBASE©, Global Health©, SCOPUS©, PsycINFO©, Sociological Abstracts©, CINAHL (Cumulative Index to Nursing and Allied Health Literature) ©, Web of Science©, and POPLine©.

Peer-reviewed conference abstracts will be searched from online publications of conference proceedings, including those of International AIDS Conference (IAC), the Conference on HIV Pathogenesis, Treatment, and Prevention, HIV Research for Prevention (HIVR4P), and the Conference on Retroviruses and Opportunistic Infections (CROI). The World Health Organization (WHO) publications database will be also searched as well as the National Library of Medicine’s Meeting Abstracts database (https://wwwcf.nlm.nih.gov/hsr_project/home_proj.cfm).

Other data sources from the gray literature will be identified through the Development Experience Clearinghouse, including national surveillance system data reports for example Demographic Health Surveys and Integrated Biological and Behavioral Surveys, as well as studies conducted by large international non-governmental organizations. The Clearinghouse will be systematically searched. These reports have been previously synthesized from global reporting databases, Global Fund grant application documents, and the gray literature [17].

Search strategies were developed based on a combination of controlled vocabulary (e.g., MeSH terms) and other keyword searches. These search strategies were adapted from existing search strategies developed for earlier systematic reviews of key populations [5, 18–20]. Multiple iterations of the search strategies were piloted in order to prioritize a highly sensitive search that captured all available data for key populations and HIV. The search strategies are made up of search terms for three independent concepts. Concept one is made up of terms for the population of interest, concept two is made up of terms for HIV, and concept three is made up of terms for violence. Each search run is a combination of concept one AND (concept two OR concept three). Detailed search strategies are available in Additional file 1.

### Inclusion and exclusion criteria

To be included in the review, data must meet the following criteria:

1. Studies of any design that include either HIV data or violence data among female sex workers, men who have sex with men, people who use drugs, transgender populations, and incarcerated populations, even if these groups are not the main focus of the study.
2. Participants in studies can be of any age, race, or ethnicity.
3. Data must be published in a peer-reviewed journal, presented as an abstract at a scientific conference, or available on the web from governmental or non-governmental sources.
4. Qualitative studies and modeling studies will be included as searchable records in the data repository, but qualitative data will be not abstracted or included in the quantitative narrative analysis.
5. Published or presented between January 1, 2006, and January 1, 2019.
6. Data from all countries and settings will be included.

The following types of studies will be excluded from the review:

1. Studies where the sample size was less than 50.
2. Studies published in languages other than English, French, and Spanish.

### Screening and selection

Titles, abstracts, citation information, and descriptor terms of citations identified through the search strategy will be screened by a team of reviewers and will be selected to move to full-text review if there is reason to believe that the above six criteria are met. Full-text articles will be obtained of all selected abstracts and the team of reviewers will conduct two independent reviews of each article to assess all full-text articles for eligibility to determine final study selection. The same set of questions will be used for full-text screening. Title/abstract and full-text review will be conducted in Covidence, a tool designed to help facilitate the systematic review process, produced in partnership with Cochrane Reviews. Differences will be resolved by a third independent reviewer.

### Data abstraction and data collection process

Data will be abstracted independently by a team of reviewers using standardized data abstraction forms in REDCap. Differences in data abstraction will be resolved using REDCap’s data comparison tool by a third, independent reviewer. Reviewers will be trained using the data collection tool on how to abstract available information from eligible articles and how to index the article in the database. While not all articles will have information for all indicators being captured in the REDCap tool, reviewers will complete the data tool based on available data. The REDCap tool outlining data capture of outcomes is available in Additional file 2.

The following information will be gathered from each included study:

- Study identification: author(s); citation; year of publication
- Study description: location, setting, population; years (period of study); study design; sample size; age range; sex and gender, if reported separately
- Outcomes (specified above)

The review and corresponding data repository will be updated regularly. The plan for updating is to run automated searches based on the same search strategies originally developed once every 6 months, following the end date of the initial review. The first update will be done in June 2019 to include articles published between January and June 2019. Data review and entry will be done on a rolling basis.

Amendments to the protocol originally published in PROSPERO include the following:

- The available data and outcomes have been more precisely specified.
- Articles published in other languages including French and Spanish will be reviewed.
- The quality assessment tool developed is a novel tool rather than a modified Downs and Black checklist.

### Quality assessments

A quality assessment tool has been developed and adapted for key populations research from the NHLBI Quality Assessment Tool for Observational Cohort and Cross-Sectional Studies [21, 22]. The tool was adapted by the study team in order to capture the essential elements of the original tool, while also ensuring applicability to our main outcomes. The tool is designed such that two independent assessors evaluate first the general study design and the study implementation and then evaluate outcome-specific data quality. The quality assessment tool is designed to assess the quality of available evidence for data points of HIV prevalence, incidence, the HIV care continuum, and population size estimates. Data points will be categorized as “good,” “fair,” or “poor” based on an evaluation of a number of criteria related to study design, study implementation, and use of appropriate analytic methods.

The quality assessment tool will be applied to available data points for 30 priority countries, selected from the larger review because they have significant bilateral and multilateral support for HIV programs. The choice of 30 priority countries is based on current donor priorities—where resources are invested, where current programs for key populations and HIV exist, and where more data are needed to inform programs. As a starting point, therefore, we wanted to focus our efforts on conducting quality assessments in countries where the assessments would be used immediately.

All articles and reports available for a specific country will be gathered and efforts will be taken to group publications based on the study of origin. Papers from the same original study will be reviewed together to evaluate the quality of the reported data emanating from the single study.

The quality assessment tool is available in Additional file 3.

### Evidence synthesis

The primary analysis will involve looking at a synthesis of the burden of HIV and the HIV treatment cascade among key populations living with HIV. We will investigate comparisons by population and by country, in particular focusing on comparisons within sub-Saharan Africa and regions within sub-Saharan Africa. We anticipate heterogeneity of the data and therefore propose a narrative synthesis. If feasible, we will consider including a meta-analysis. Secondary analyses will involve looking at reporting of pre-exposure prophylaxis (PrEP) use and PrEP uptake among those who are HIV negative. Similarly, this will involve comparisons across populations and countries and will involve a narrative synthesis. Additional secondary analyses involving prevention indicators (engaged in HIV testing, knowledge of HIV prevention, condom availability, consistent condom use), population size estimates, experienced violence (physical, sexual, intimate partner), and engagement with healthcare systems for key populations may be proposed at a later date.

### Dissemination

In response to limitations and challenges in using data from previous reviews, we determined there will be added value to capture all of the data reviewed and included for this review in a database that is accessible and updatable. The results will therefore be used to generate a data repository for key populations that will be integrated into an online, graphical interface that will display global HIV data synthesized during the systematic review. The online global map will visually display all available data, will allow for comparison of key HIV statistics among different countries, will provide country dashboard pages that highlight key information, and will allow for datasets to be downloaded by researchers, government officials, community-based organizations, members of the community, and the general public.

## Discussion

Despite evidence of the importance and overall impact of prioritizing key populations in implementing effective and efficient HIV responses, many countries have limited current data or no data characterizing the unmet needs of key populations [9–13, 23, 24]. Where data are available, they are often not used in key documents and policy decisions, despite an international call to further evidence-based policies [14]. Systematic reviews conducted in the past among key populations have comprehensively identified where data exist and synthesized available data to provide estimates on a range of indicators, including HIV prevalence, HIV incidence, and population size estimates [5, 6, 14, 18, 19, 25–30]. However, with rapidly evolving methods for estimating population size and HIV transmission dynamics, these estimates have become dated both in terms of age of data and their usability by key stakeholders. Moreover, given persistent stigma and ever-changing laws and policies related to the criminalization of key populations including laws focused on same-sex practices, sex work, gender identity, and injection drug use, the need for current and local level estimates is crucial for key populations [31]. To this end, the current review, online platform, and plans for ongoing and regular updates will make responding to changing contexts and environments more feasible in real-time.

Past reviews have synthesized data to produce pooled estimates at the national or global level. This level of analysis is helpful for understanding the unmet needs for key populations, but accessing and critically assessing individual data points can help better inform the decision of the utility of these data for informing policy. The current review is intended to be flexible, sustainable, and relevant over time, with updates to the global interactive tool scheduled to happen on a regular basis. Key stakeholders will have the ability to make data requests that specify the populations, countries, or indicators in which they have the interest or need. These data pulls will contain key study design information where available and quality assessments so that those using the data have the relevant information they need to make informed decisions about data use.

We have identified several challenges in designing and conducting this updatable systematic review of global key population data. First, capturing information from a large pool of sources from disparate fields of study, among different populations, and in different countries required us to make decisions on what information we could feasibly capture. As an example, consistent condom use is often an important indicator or predictor for assessing level of sexual transmission of HIV, risk for other STIs, partner dynamics, and uptake of interventions designed to improve condom use. Consistent condom use, however, is not systematically defined. How a study or report defines consistent condom use can vary in terms of time period [e.g., condoms used during the last act, the last week, the last 30 days], frequency [e.g., always/sometimes/never, number of times], and partner type [e.g., with all partners, disaggregated by clients/regular/casual], etc. In addition, there are differences in how much is specified related to what “condom use” even means, that is was one “ever” used, was it used for the “whole duration of the act”, was it “reused.” We wanted to capture consistent condom use but had to account for differences in reporting. To be able to capture consistent condom use then, we decided to capture the proportion who reported consistent condom use however the study or report defined it, but to have an additional indicator allowing the data entrant to specify what exactly “consistent condom use” meant for each data point. Another challenge we identified was dealing with multiple studies or reports published from the same study. Because our unit of analysis in this systematic review was the data source, it was important to determine a strategy to account for duplication of certain data points. To do this, we created a cohort identifier based on key information that would apply to the study itself rather than the publication or report in order to have the ability to later identify which sources were actually referring to the same data point. Including all of the estimates would bias our results towards those studies with the most publications. Finally, in order to create a user-friendly online resource for collating and displaying global HIV data for key populations, we needed to make decisions on how to visualize data on a range of indicators, including prevalence, incidence, the treatment cascade, and population size estimates. There are multiple levels to the data that are being synthesized: key population group, country, and indicator of interest, and creating a system that would not only display the available data in a clear, understandable way, but also be flexible and updatable, continues to be a challenge. With the support of our web development team and the infrastructure of Carto, we have been able to accommodate these different levels of data and allow for ease of use. We chose to use an interactive global map that allows the user to select key indicators to geographically display, provides country-specific dashboards with key information and indicators highlighted for different key population groups, and creates data visualization graphs showing changes in key indicators over time and comparisons across countries. The value in conducting this large systematic review, with a range of indicators, is that it will allow us to go beyond standard single population systematic reviews of key population groups, giving us the ability to assess overlap of populations (e.g., female sex worker who injects drugs) and make comparisons between groups on key indicators. This will also allow for comprehensive identification of data gaps or unmet needs among key populations and prioritization of funding for individual populations based on the context-specific needs within a single country or region. Findings from this systematic review and the development of the enhanced graphical interface to display data will help ensure that available epidemiologic data related to key populations can be more effectively used in real-time to inform HIV-related policies and programs.

## Additional files

Additional file 1: Provides detailed search strategies by database. (DOCX 27 kb)

Additional file 2: Provides the data collection tool for abstraction of a range of study specific details and HIV and violence related indicators for key populations. (PDF 321 kb)

Additional file 3: Provides the quality assessment tool used to evaluate data points for prevalence and incidence of HIV, the treatment cascade, and population size estimates. (DOCX 32 kb)

## Abbreviations

AIDS: Acquired immunodeficiency syndrome
COPs: Country Operational Plans
CROI: Conference on Retroviruses and Opportunistic Infections
HIV: Human immunodeficiency virus
HIVR4P: HIV Research for Prevention
IAC: International AIDS Conference
MeSH: Medical subject headings
MOT: Modes of transmission
NHLBI: National Heart, Lung, and Blood Institute
PEPFAR: President’s Emergency Plan for AIDS Relief
PrEP: Pre-exposure prophylaxis
STIs: Sexually transmitted infections
WHO: World Health Organization
