# Supplementary material for: HIV-related data among key populations to inform evidence-based responses: protocol of a systematic review
Source: Syst Rev. 2018 Dec 3;7:220. doi: 10.1186/s13643-018-0894-3 (PMC6278072; doi:10.1186/s13643-018-0894-3)
Supplement: Supplementary file 1 — Provides detailed search strategies by database. (DOCX 27 kb) [file 13643_2018_894_MOESM1_ESM.docx]

**PubMed**

**Concept 1: Female sex workers**

"Prostitution"[MeSH] OR "Sex Workers"[MeSH] OR sex work*[tw] OR prostitut*[tw] OR "commercial sex"[tw] OR "transactional sex"[tw] OR "SW"[tw] OR "FSW"[tw] OR "CSW"[tw] OR "sex trade"[tw] OR "trade sex"[tw]

**Concept 1: Men who have sex with men**

"Homosexuality, Male"[Mesh] OR "gay men"[tw] OR "gay man"[tw] OR gay male*[tw] OR homosexual*[tw] OR "MSM"[tw] OR "men who have sex with men"[tw] OR "males who have sex with males"[tw] OR "bisexual men"[tw] OR "bisexual man"[tw] OR bisexual male*[tw]

**Concept 1: People who use drugs**

"Drug Users"[Mesh] OR "Substance Abuse, Intravenous"[Mesh] OR "drug use"[tw] OR "drug user"[tw] OR "drug users"[tw] OR drug abuse*[tw] OR substance use*[tw] OR substance abuse*[tw] OR people who inject drug*[tw] OR people who use drug*[tw] OR "IVDU"[tw] OR "IDU"[tw] OR "PWUD"[tw] OR "PWID"[tw] OR "drug usage"[tw]

**Concept 1: Transgender people**

"Transgender Persons"[Mesh] OR transgender*[tw] OR transsexual*[tw] OR transvest*[tw] OR travesti*[tw] OR cross dress*[tw] OR "koti"[tw] OR "hijra"[tw] OR "mahu"[tw] OR "waria"[tw] OR "katoey"[tw] OR "berdache"[tw] OR "male to female transgender"[tw] OR "MTF"[tw] OR transmascul*[tw]

**Concept 1: Incarcerated populations**

"Prisoners"[Mesh] OR incarcerat*[tw] OR prison*[tw]

**Concept 2: HIV**

"HIV"[Mesh] OR "Acquired Immunodeficiency Syndrome"[Mesh] OR "HIV Infections"[Mesh] OR human immunodeficiency virus*[tw] OR acquired immunodeficiency syndrome*[tw] OR HIV*[tw] OR "AIDS"[tw] OR HIV1*[tw] OR HIV2*[tw]

**Concept 3: Violence**

"Violence"[Mesh] OR "Domestic Violence"[Mesh] OR "Workplace Violence"[Mesh] OR "Crime Victims"[Mesh] OR "Battered Women"[Mesh] OR "Human Rights Abuses"[Mesh] OR "Sex Offenses"[Mesh] OR "Homicide"[Mesh] OR "Coercion"[Mesh] OR "Sexual Harassment"[Mesh] OR "Torture"[Mesh] OR "Spouse Abuse"[Mesh] OR "Rape"[Mesh] OR violen*[tw] OR crime*[tw] OR offense*[tw] OR abuse*[tw] OR victim*[tw] OR rape*[tw] OR assault*[tw] OR batter*[tw] OR extort*[tw] OR blackmail*[tw] OR intimidat*[tw] OR exploit*[tw] OR "IPV"[tw] OR "IPSV"[tw]

**Date range**

("2010/MM/DD"[PDAT] : "3000/MM/DD"[PDAT])

**(CONCEPT 2 OR CONCEPT 3) AND CONCEPT 1=**

**Embase – DDMMYY**

**Concept 1: Female sex workers**

'prostitution'/exp OR 'transactional sex'/exp OR ('sex work*' OR 'prostitut*' OR 'commercial sex' OR 'transactional sex' OR 'SW' OR 'FSW' OR 'CSW' OR 'sex trade' OR 'trade sex'):ab,ti

**Concept 1: Men who have sex with men**

'men who have sex with men'/exp OR 'homosexual male'/exp OR 'male homosexuality'/exp OR ('gay men' OR 'gay man' OR 'gay male*' OR 'homosexual*' OR 'MSM' OR 'men who have sex with men' OR 'males who have sex with males' OR 'bisexual men' OR 'bisexual man' OR 'bisexual male*'):ab,ti

**Concept 1: People who use drugs**

'intravenous drug abuse'/exp OR ('drug use' OR 'drug user' OR 'drug users' OR 'drug abuse*' OR 'substance use*' OR 'substance abuse*' OR 'people who inject drug*' OR 'people who use drug*' OR 'IVDU' OR 'IDU' OR 'PWUD' OR 'PWID' OR 'drug usage'):ab,ti

**Concept 1: Transgender people**

'transgender'/exp OR 'female to male transgender'/exp OR ('transgender*' OR 'transsexual*' OR 'transvest*' OR 'travesti*' OR 'cross dress*' OR 'koti' OR 'hijra' OR 'mahu' OR 'waria' OR 'katoey' OR 'berdache' OR 'male to female transgender' OR 'MTF' OR 'transmascul*'):ab,ti

**Concept 1: Incarcerated populations**

'prisoner'/exp OR ('incarcerat*' OR 'prison*'):ab,ti

**Concept 2: HIV**

'Human immunodeficiency virus'/exp OR 'acquired immune deficiency syndrome'/exp OR ('human immunodeficiency virus*' OR 'acquired immunodeficiency syndrome*' OR 'HIV*' OR 'AIDS' OR 'HIV1*' OR 'HIV2*'):ab,ti

**Concept 3: Violence**

'violence'/exp OR 'assault'/exp OR 'battering'/exp OR 'dating violence'/exp OR 'domestic violence'/exp OR 'partner violence'/exp OR 'gender based violence'/exp OR 'homicide'/exp OR 'physical violence'/exp OR 'sexual violence'/exp OR 'workplace violence'/exp OR 'torture'/exp OR ('violen*' OR 'crime*' OR 'offense*' OR 'abuse*' OR 'victim*' OR 'rape*' OR 'assault*' OR 'batter*' OR 'extort*' OR 'blackmail*' OR 'intimidat*' OR 'exploit*' OR 'IPV' OR 'IPSV'):ab,ti

DATE: (2006:py OR 2007:py OR 2008:py OR 2009:py OR 2010:py OR 2011:py OR 2012:py OR 2013:py OR 2014:py OR 2015:py OR 2016:py)

**(CONCEPT 2 OR CONCEPT 3) AND CONCEPT 1=**

**Global Health – DDMMYY**

**Concept 1: Female sex workers**

exp prostitution/ OR exp sex workers OR ("sex work*" OR "prostitut*" OR "commercial sex" OR "transactional sex" OR "SW" OR "FSW" OR "CSW" OR "sex trade" OR "trade sex").mp.

**Concept 1: Men who have sex with men**

exp men who have sex with men/ OR exp homosexuality/ OR ("gay men" OR "gay man" OR "gay male*" OR "homosexual*" OR "MSM" OR "men who have sex with men" OR "males who have sex with males" OR "bisexual men" OR "bisexual man" OR "bisexual male*").mp.

**Concept 1: People who use drugs**

exp drug users/ OR exp substance abuse/ OR ("drug use" OR "drug user" OR "drug users" OR "drug abuse*" OR "substance use*" OR "substance abuse*" OR "people who inject drug*" OR "people who use drug*" OR "IVDU" OR "IDU" OR "PWUD" OR "PWID" OR "drug usage").mp.

**Concept 1: Transgender people**

("transgender*" OR "transsexual*" OR "transvest*" OR "travesti*" OR "cross dress*" OR "koti" OR "hijra" OR "mahu" OR "waria" OR "katoey" OR "berdache" OR "male to female transgender" OR "MTF" OR "transmascul*").mp.

**Concept 1: Incarcerated populations**

exp prisoners/ OR (incarcerat* OR prison*).mp

**Concept 2: HIV**

exp human immunodeficiency viruses/ OR exp acquired immune deficiency syndrome/ OR ("human immunodeficiency virus*" OR "acquired immunodeficiency syndrome*" OR "HIV*" OR "AIDS" OR "HIV1*" OR "HIV2*").mp.

**Concept 3: Violence**

|  |
| --- |

exp domestic violence/ OR exp rape/ OR exp sexual assault/ OR (violen* OR crime* OR offense* OR abuse* OR victim* OR rape* OR assault* OR batter* OR extort* OR blackmail* OR intimidat* OR exploit* OR "IPV" OR "IPSV").mp.

**(CONCEPT 2 OR CONCEPT 3) AND CONCEPT 1=**

**SCOPUS – DDMMYY**

**Concept 1: Female sex workers**

TITLE-ABS-KEY("sex work*" OR "prostitut*" OR "commercial sex" OR "transactional sex" OR "SW" OR "FSW" OR "CSW" OR "sex trade" OR "trade sex")

**Concept 1: Men who have sex with men**

TITLE-ABS-KEY("gay men" OR "gay man" OR "gay male*" OR "homosexual*" OR "MSM" OR "men who have sex with men" OR "males who have sex with males" OR "bisexual men" OR "bisexual man" OR "bisexual male*")

**Concept 1: People who use drugs**

TITLE-ABS-KEY("drug use" OR "drug user" OR "drug users" OR "drug abuse*" OR "substance use*" OR "substance abuse*" OR "people who inject drug*" OR "people who use drug*" OR "IVDU" OR "IDU" OR "PWUD" OR "PWID" OR "drug usage")

**Concept 1: Transgender people**

TITLE-ABS-KEY("transgender*" OR "transsexual*" OR "transvest*" OR "travesti*" OR "cross dress*" OR "koti" OR "hijra" OR "mahu" OR "waria" OR "katoey" OR "berdache" OR "male to female transgender" OR "MTF" OR "transmascul*")

**Concept 1: Incarcerated populations**

TITLE-ABS-KEY("incarcerat*" OR "prison*")

**Concept 2: HIV**

TITLE-ABS-KEY("human immunodeficiency virus*" OR "acquired immunodeficiency syndrome*" OR "HIV*" OR "AIDS" OR "HIV1*" OR "HIV2*")

**Concept 3: Violence**

TITLE-ABS-KEY("violen*" OR "crime*" OR "offense*" OR "abuse*" OR "victim*" OR "rape*" OR "assault*" OR "batter*" OR "extort*" OR "blackmail*" OR "intimidat*" OR "exploit*" OR "IPV" OR "IPSV")

PUBYEAR is 2006 OR PUBYEAR is 2007 OR PUBYEAR is 2008 OR PUBYEAR is 2009 OR PUBYEAR is 2010 OR PUBYEAR is 2011 OR PUBYEAR is 2012 OR PUBYEAR is 2013 OR PUBYEAR is 2014 OR PUBYEAR is 2015 OR PUBYEAR is 2016 OR PUBYEAR is 2017

**(CONCEPT 2 OR CONCEPT 3) AND CONCEPT 1=**

**PsycINFO – DDMMYY**

**Concept 1: Female sex workers**

DE "Prostitution" OR TI ( "sex work*" OR "prostitut*" OR "commercial sex" OR "transactional sex" OR "SW" OR "FSW" OR "CSW" OR "sex trade" OR "trade sex" ) OR AB ( "sex work*" OR "prostitut*" OR "commercial sex" OR "transactional sex" OR "SW" OR "FSW" OR "CSW" OR "sex trade" OR "trade sex" ) OR KW ( "sex work*" OR "prostitut*" OR "commercial sex" OR "transactional sex" OR "SW" OR "FSW" OR "CSW" OR "sex trade" OR "trade sex" )

**Concept 1: Men who have sex with men**

DE "Same Sex Intercourse" OR DE "Male Homosexuality" OR TI ( "gay men" OR "gay man" OR "gay male*" OR "homosexual*" OR "MSM" OR "men who have sex with men" OR "males who have sex with males" OR "bisexual men" OR "bisexual man" OR "bisexual male*" ) OR AB ( "gay men" OR "gay man" OR "gay male*" OR "homosexual*" OR "MSM" OR "men who have sex with men" OR "males who have sex with males" OR "bisexual men" OR "bisexual man" OR "bisexual male*" ) OR KW ( "gay men" OR "gay man" OR "gay male*" OR "homosexual*" OR "MSM" OR "men who have sex with men" OR "males who have sex with males" OR "bisexual men" OR "bisexual man" OR "bisexual male*" )

**Concept 1: People who use drugs**

DE "Drug Abuse" OR TI ( "drug use" OR "drug user" OR "drug users" OR "drug abuse*" OR "substance use*" OR "substance abuse*" OR "people who inject drug*" OR "people who use drug*" OR "IVDU" OR "IDU" OR "PUD" OR "PWUD" OR "PWID" OR "drug usage" ) OR AB ( "drug use" OR "drug user" OR "drug users" OR "drug abuse*" OR "substance use*" OR "substance abuse*" OR "people who inject drug*" OR "people who use drug*" OR "IVDU" OR "IDU" OR "PUD" OR "PWUD" OR "PWID" OR "drug usage" ) OR KW ( "drug use" OR "drug user" OR "drug users" OR "drug abuse*" OR "substance use*" OR "substance abuse*" OR "people who inject drug*" OR "people who use drug*" OR "IVDU" OR "IDU" OR "PUD" OR "PWUD" OR "PWID" OR "drug usage" )

**Concept 1: Transgender people**

DE "Transgender" OR TI ( "transgender*" OR "transsexual*" OR "transvest*" OR "travesti*" OR "cross dress*" OR "koti" OR "hijra" OR "mahu" OR "waria" OR "katoey" OR "berdache" OR "male to female transgender" OR "MTF" OR "transmascul*" ) AB ( "transgender*" OR "transsexual*" OR "transvest*" OR "travesti*" OR "cross dress*" OR "koti" OR "hijra" OR "mahu" OR "waria" OR "katoey" OR "berdache" OR "male to female transgender" OR "MTF" OR "transmascul*" ) KW ("transgender*" OR "transsexual*" OR "transvest*" OR "travesti*" OR "cross dress*" OR "koti" OR "hijra" OR "mahu" OR "waria" OR "katoey" OR "berdache" OR "male to female transgender" OR "MTF" OR "transmascul*" )

**Concept 1: Incarcerated populations**

DE "Prisoners" OR TI ( "incarcerat*" OR "prison*" ) OR AB ( "incarcerat*" OR "prison*" ) OR KW ( "incarcerat*" OR "prison*" )

**Concept 2: HIV**

DE "HIV" OR DE "AIDS" OR TI ( "human immunodeficiency virus*" OR "acquired immunodeficiency syndrome*" OR "HIV*" OR "AIDS" "HIV1*" OR "HIV2*" ) OR AB ( "human immunodeficiency virus*" OR "acquired immunodeficiency syndrome*" OR "HIV*" OR "AIDS" "HIV1*" OR "HIV2*" ) OR KW ( "human immunodeficiency virus*" OR "acquired immunodeficiency syndrome*" OR "HIV*" OR "AIDS" "HIV1*" OR "HIV2*" )

**Concept 3: Violence**

DE "Violence" OR DE "Domestic Violence" OR DE "Intimate Partner Violence" OR DE "Violent Crime" OR DE "Workplace Violence" OR DE "Rape" OR DE "Acquaintance Rape" OR DE "Sex Offenses" OR TI ( "violen*" OR "crime*" OR "offense*" OR "abuse*" OR "victim*" OR "rape*" OR "assault*" OR "batter*" OR "extort*" OR "blackmail*" OR "intimidat*" OR "exploit*" OR "IPV" OR "IPSV" ) OR AB ("violen*" OR "crime*" OR "offense*" OR "abuse*" OR "victim*" OR "rape*" OR "assault*" OR "batter*" OR "extort*" OR "blackmail*" OR "intimidat*" OR "exploit*" OR "IPV" OR "IPSV" ) OR KW ( "violen*" OR "crime*" OR "offense*" OR "abuse*" OR "victim*" OR "rape*" OR "assault*" OR "batter*" OR "extort*" OR "blackmail*" OR "intimidat*" OR "exploit*" OR "IPV" OR "IPSV" )

**(CONCEPT 2 OR CONCEPT 3) AND CONCEPT 1=**

**Sociological Abstracts – DDMMYY**

**Concept 1: Female sex workers**

SU.EXACT("Prostitution") OR [ti("sex work*" OR "prostitut*" OR "commercial sex" OR "transactional sex" OR "SW" OR "FSW" OR "CSW" OR "sex trade" OR "trade sex") OR ab("sex work*" OR "prostitut*" OR "commercial sex" OR "transactional sex" OR "SW" OR "FSW" OR "CSW" OR "sex trade" OR "trade sex") OR su("sex work*" OR "prostitut*" OR "commercial sex" OR "transactional sex" OR "SW" OR "FSW" OR "CSW" OR "sex trade" OR "trade sex")](http://search.proquest.com/recentsearches.recentsearchtabview.recentsearchesgridview.scrolledrecentsearchlist.checkdbssearchlink:rerunsearch/8326F199315B48B6PQ/None?site=socabs&t:ac=RecentSearches)

**Concept 1: Men who have sex with men**

SU.EXACT("Homosexuality") OR ti("gay men" OR "gay man" OR "gay male*" OR "homosexual*" OR "MSM" OR "men who have sex with men" OR "males who have sex with males" OR "bisexual men" OR "bisexual man" OR "bisexual male*") OR ab("gay men" OR "gay man" OR "gay male*" OR "homosexual*" OR "MSM" OR "men who have sex with men" OR "males who have sex with males" OR "bisexual men" OR "bisexual man" OR "bisexual male*") OR su("gay men" OR "gay man" OR "gay male*" OR "homosexual*" OR "MSM" OR "men who have sex with men" OR "males who have sex with males" OR "bisexual men" OR "bisexual man" OR "bisexual male*")

**Concept 1: People who use drugs**

SU.EXACT("Drug Abuse") OR ti("drug use" OR "drug user" OR "drug users" OR "drug abuse*" OR "substance use*" OR "substance abuse*" OR "people who inject drug*" OR "people who use drug*" OR "IVDU" OR "IDU" OR "PUD" OR "PWUD" OR "PWID" OR "drug usage") OR ab("drug use" OR "drug user" OR "drug users" OR "drug abuse*" OR "substance use*" OR "substance abuse*" OR "people who inject drug*" OR "people who use drug*" OR "IVDU" OR "IDU" OR "PUD" OR "PWUD" OR "PWID" OR "drug usage") OR su("drug use" OR "drug user" OR "drug users" OR "drug abuse*" OR "substance use*" OR "substance abuse*" OR "people who inject drug*" OR "people who use drug*" OR "IVDU" OR "IDU" OR "PUD" OR "PWUD" OR "PWID" OR "drug usage")

**Concept 1: Transgender people**

SU.EXACT("Transvestism") OR ti("transgender*" OR "transsexual*" OR "transvest*" OR "travesti*" OR "cross dress*" OR "koti" OR "hijra" OR "mahu" OR "waria" OR "katoey" OR "berdache" OR "male to female transgender" OR "MTF" OR "transmascul*") OR ab("transgender*" OR "transsexual*" OR "transvest*" OR "travesti*" OR "cross dress*" OR "koti" OR "hijra" OR "mahu" OR "waria" OR "katoey" OR "berdache" OR "male to female transgender" OR "MTF" OR "transmascul*") OR su("transgender*" OR "transsexual*" OR "transvest*" OR "travesti*" OR "cross dress*" OR "koti" OR "hijra" OR "mahu" OR "waria" OR "katoey" OR "berdache" OR "male to female transgender" OR "MTF" OR "transmascul*")

**Concept 1: Incarcerated populations**

SU.EXACT("Prisoners") OR ti("incarcerat*" OR "prison*") OR ab("incarcerat*" OR "prison*") OR su("incarcerat*" OR "prison*")

**Concept 2: HIV**

SU.EXACT("Acquired Immune Deficiency Syndrome") OR ti("human immunodeficiency virus*" OR "acquired immunodeficiency syndrome*" OR "HIV*" OR "AIDS" OR "HIV1*" OR "HIV2*") OR ab("human immunodeficiency virus*" OR "acquired immunodeficiency syndrome*" OR "HIV*" OR "AIDS" OR "HIV1*" OR "HIV2*") OR su("human immunodeficiency virus*" OR "acquired immunodeficiency syndrome*" OR "HIV*" OR "AIDS" OR "HIV1*" OR "HIV2*")

**Concept 3: Violence**

SU.EXACT("Sexual Assault") OR SU.EXACT("Violence") OR SU.EXACT("Family Violence") OR ti("violen*" OR "crime*" OR "offense*" OR "abuse*" OR "victim*" OR "rape*" OR "assault*" OR "batter*" OR "extort*" OR "blackmail*" OR "intimidat*" OR "exploit*" OR "IPV" OR "IPSV") OR ab("violen*" OR "crime*" OR "offense*" OR "abuse*" OR "victim*" OR "rape*" OR "assault*" OR "batter*" OR "extort*" OR "blackmail*" OR "intimidat*" OR "exploit*" OR "IPV" OR "IPSV") OR su("violen*" OR "crime*" OR "offense*" OR "abuse*" OR "victim*" OR "rape*" OR "assault*" OR "batter*" OR "extort*" OR "blackmail*" OR "intimidat*" OR "exploit*" OR "IPV" OR "IPSV")

**(CONCEPT 2 OR CONCEPT 3) AND CONCEPT 1=**

**CINAHL – DDMMYY**

**Concept 1: Female sex workers**

MH "Prostitution" OR TI ( "sex work*" OR "prostitut*" OR "commercial sex" OR "transactional sex" OR "SW" OR "FSW" OR "CSW" OR "sex trade" OR "trade sex" ) OR AB ( "sex work*" OR "prostitut*" OR "commercial sex" OR "transactional sex" OR "SW" OR "FSW" OR "CSW" OR "sex trade" OR "trade sex" ) OR SU ( "sex work*" OR "prostitut*" OR "commercial sex" OR "transactional sex" OR "SW" OR "FSW" OR "CSW" OR "sex trade" OR "trade sex" )

**Concept 1: Men who have sex with men**

MH "Homosexuals, Male" OR TI ( "gay men" OR "gay man" OR "gay male*" OR "homosexual*" OR "MSM" OR "men who have sex with men" OR "males who have sex with males" OR "bisexual men" OR "bisexual man" OR "bisexual male*" ) OR AB ( "gay men" OR "gay man" OR "gay male*" OR "homosexual*" OR "MSM" OR "men who have sex with men" OR "males who have sex with males" OR "bisexual men" OR "bisexual man" OR "bisexual male*" ) OR KW ( "gay men" OR "gay man" OR "gay male*" OR "homosexual*" OR "MSM" OR "men who have sex with men" OR "males who have sex with males" OR "bisexual men" OR "bisexual man" OR "bisexual male*" )

**Concept 1: People who use drugs**

MH "Substance Abuse" OR TI ( "drug use" OR "drug user" OR "drug users" OR "drug abuse*" OR "substance use*" OR "substance abuse*" OR "people who inject drug*" OR "people who use drug*" OR "IVDU" OR "IDU" OR "PUD" OR "PWUD" OR "PWID" OR "drug usage" ) OR AB ( "drug use" OR "drug user" OR "drug users" OR "drug abuse*" OR "substance use*" OR "substance abuse*" OR "people who inject drug*" OR "people who use drug*" OR "IVDU" OR "IDU" OR "PUD" OR "PWUD" OR "PWID" OR "drug usage" ) OR KW ( "drug use" OR "drug user" OR "drug users" OR "drug abuse*" OR "substance use*" OR "substance abuse*" OR "people who inject drug*" OR "people who use drug*" OR "IVDU" OR "IDU" OR "PUD" OR "PWUD" OR "PWID" OR "drug usage" )

**Concept 1: Transgender people**

MH "Transgendered persons" OR TI ( "transgender*" OR "transsexual*" OR "transvest*" OR "travesti*" OR "cross dress*" OR "koti" OR "hijra" OR "mahu" OR "waria" OR "katoey" OR "berdache" OR "male to female transgender" OR "MTF" OR "transmascul*" ) AB ( "transgender*" OR "transsexual*" OR "transvest*" OR "travesti*" OR "cross dress*" OR "koti" OR "hijra" OR "mahu" OR "waria" OR "katoey" OR "berdache" OR "male to female transgender" OR "MTF" OR "transmascul*" ) KW ("transgender*" OR "transsexual*" OR "transvest*" OR "travesti*" OR "cross dress*" OR "koti" OR "hijra" OR "mahu" OR "waria" OR "katoey" OR "berdache" OR "male to female transgender" OR "MTF" OR "transmascul*" )

**Concept 1: Incarcerated populations**

MH "Prisoners" OR TI ( "incarcerat*" OR "prison*" ) OR AB ( "incarcerat*" OR "prison*" ) OR KW ( "incarcerat*" OR "prison*" )

**Concept 2: HIV**

MH "Human Immunodeficiency Virus" OR MH "Acquired Immunodeficiency Syndrome" OR TI ( "human immunodeficiency virus*" OR "acquired immunodeficiency syndrome*" OR "HIV*" OR "AIDS" "HIV1*" OR "HIV2*" ) OR AB ( "human immunodeficiency virus*" OR "acquired immunodeficiency syndrome*" OR "HIV*" OR "AIDS" "HIV1*" OR "HIV2*" ) OR KW ( "human immunodeficiency virus*" OR "acquired immunodeficiency syndrome*" OR "HIV*" OR "AIDS" "HIV1*" OR "HIV2*" )

**Concept 3: Violence**

MH "Violence" OR MH "Workplace Violence" OR MH "Domestic Violence" OR MH "Intimate Partner Violence" OR MH "Rape" OR TI ( "violen*" OR "crime*" OR "offense*" OR "abuse*" OR "victim*" OR "rape*" OR "assault*" OR "batter*" OR "extort*" OR "blackmail*" OR "intimidat*" OR "exploit*" OR "IPV" OR "IPSV" ) OR AB ("violen*" OR "crime*" OR "offense*" OR "abuse*" OR "victim*" OR "rape*" OR "assault*" OR "batter*" OR "extort*" OR "blackmail*" OR "intimidat*" OR "exploit*" OR "IPV" OR "IPSV" ) OR KW ( "violen*" OR "crime*" OR "offense*" OR "abuse*" OR "victim*" OR "rape*" OR "assault*" OR "batter*" OR "extort*" OR "blackmail*" OR "intimidat*" OR "exploit*" OR "IPV" OR "IPSV" )

**(CONCEPT 2 OR CONCEPT 3) AND CONCEPT 1=**

**Web of Science – DDMMYY**

**Concept 1: Female sex workers**

TS=("sex work*" OR "prostitut*" OR "commercial sex" OR "transactional sex" OR "SW" OR "FSW" OR "CSW" OR "sex trade" OR "trade sex")

**Concept 1: Men who have sex with men**

TS=("gay men" OR "gay man" OR "gay male*" OR "homosexual*" OR "MSM" OR "men who have sex with men" OR "males who have sex with males" OR "bisexual men" OR "bisexual man" OR "bisexual male*")

**Concept 1: People who use drugs**

TS=("drug use" OR "drug user" OR "drug users" OR "drug abuse*" OR "substance use*" OR "substance abuse*" OR "people who inject drug*" OR "people who use drug*" OR "IVDU" OR "IDU" OR "PUD" OR "PWUD" OR "PWID" OR "drug usage")

**Concept 1: Transgender people**

TS=("transgender*" OR "transsexual*" OR "transvest*" OR "travesti*" OR "cross dress*" OR "koti" OR "hijra" OR "mahu" OR "waria" OR "katoey" OR "berdache" OR "male to female transgender" OR "MTF" OR "transmascul*")

**Concept 1: Incarcerated populations**

TS=("incarcerat*" OR "prison*")

**Concept 2: HIV**

TS=("human immunodeficiency virus*" OR "acquired immunodeficiency syndrome*" OR "HIV*" OR "AIDS" OR "HIV1*" OR "HIV2*")

**Concept 3: Violence**

TS=("violen*" OR "crime*" OR "offense*" OR "abuse*" OR "victim*" OR "rape*" OR "assault*" OR "batter*" OR "extort*" OR "blackmail*" OR "intimidat*" OR "exploit*" OR "IPV" OR "IPSV")

Date Limit:

PY=2006-2016 (see if you can leave off the 2016)

**(CONCEPT 2 OR CONCEPT 3) AND CONCEPT 1=**

**POPLine – DDMMYY (no need for field tags)**

**Concept 1: Female sex workers**

"Sex workers" OR "sex work*" OR "prostitut*" OR "commercial sex" OR "transactional sex" OR "SW" OR "FSW" OR "CSW" OR "sex trade" OR "trade sex"

**Concept 1: Men who have sex with men**

"Men having sex with men" OR "homosexuals" OR "gay men" OR "gay man" OR "gay male*" OR "homosexual*" OR "MSM" OR "men who have sex with men" OR "males who have sex with males" OR "bisexual men" OR "bisexual man" OR "bisexual male*"

**Concept 1: People who use drugs**

"Drug Use and Abuse" OR "IV drug users" OR "drug use*" OR "drug abuse*" OR "substance use*" OR "substance abuse*" OR "people who inject drug*" OR "people who use drug*" OR "IVDU" OR "IDU" OR "PUD" OR "PWUD" OR "PWID" OR "drug usage"

**Concept 1: Transgender people**

"transgender*" OR "transsexual*" OR "transvest*" OR "travesti*" OR "cross dress*" OR "koti" OR "hijra" OR "mahu" OR "waria" OR "katoey" OR "berdache" OR "male to female transgender" OR "MTF" OR "transmascul*"

**Concept 1: Incarcerated populations**

"prisoners" OR "incarcerat*" OR "prison*"

**Concept 2: HIV**

"HIV" OR "human immunodeficiency virus*" OR "acquired immunodeficiency syndrome*" OR "HIV*" OR "AIDS" OR "HIV1*" OR "HIV2*"

**Concept 3: Violence**

"Violence" OR "Domestic Violence" OR "Rape" OR "Homicide" OR "Sexual Abuse" OR "Sexual Harassment" OR "violen*" OR "crime*" OR "offense*" OR "abuse*" OR "victim*" OR "rape*" OR "assault*" OR "batter*" OR "extort*" OR "blackmail*" OR "intimidat*" OR "exploit*" OR "IPV" OR "IPSV"

**(CONCEPT 2 OR CONCEPT 3) AND CONCEPT 1=**
