# Supplementary material for: HIV-related data among key populations to inform evidence-based responses: protocol of a systematic review
Source: Syst Rev. 2018 Dec 3;7:220. doi: 10.1186/s13643-018-0894-3 (PMC6278072; doi:10.1186/s13643-018-0894-3)
Supplement: Supplementary file 2 — Provides the data collection tool for abstraction of a range of study specific details and HIV and violence related indicators for key populations. (PDF 321 kb) [file 13643_2018_894_MOESM2_ESM.pdf]

KEY POPULATIONS DATA REPOSITORY

Unique ID [XXX-XXX-XXX-##] __________________________________

First three letters of first author last name

Last three letters of journal

Last three letters of of title

Year

(OR "REPORT", 3 letter country abbreviation, year)

e.g. REPORTAFG11

OVERALL STUDY DETAILS

Article file upload

Web link __________________________________

Title __________________________________

First name (first author) __________________________________

Last name (first author) __________________________________

Year of publication __________________________________

Cohort ID (##-XXX-XXX-#####) __________________________________

YEAR OF PUB (##)
POPULATION (XXX) [FSW, MSM, PWU, TRA, INC]
COUNTRY ABBREV (XXX) or "MUL"
SAMPLES SIZE (#####)

Journal name (full)/Source __________________________________

Primary focus on quantitative data?
◯ Quantitative
◯ Qualitative
◯ Modeling

Type of study/document
◯ Cross-sectional
◯ Longitudinal/cohort
◯ RCT
◯ Review/Systematic review
◯ Other

CROSS-SECTIONAL

Cross-sectional (Total sample size) ________________________

Cross-sectional (Year study was conducted) ________________________

Cross-sectional (Recruitment Method)
◯ Census
◯ Probability sampling
◯ Respondent driven sampling
◯ Time-Location Sampling/Venue-based sampling
◯ Snowball sampling
◯ Clinic/Facility-based sampling
◯ Outreach sampling
◯ Other

Specify (if other) ________________________

Cross-sectional (Notes) ______________________________

LONGITUDINAL

Longitudinal (Total sample size) ________________________

Longitudinal (Year of study start) ________________________

Longitudinal (Year of study end) ________________________

Longitudinal (Frequency of study visits)
◯ Daily
◯ Weekly
◯ Biweekly
◯ Monthly
◯ Every 3 months
◯ Every 6 months
◯ Annual

Longitudinal (Recruitment method)
◯ Census
◯ Probability sampling
◯ Respondent driven sampling
◯ Time-Location Sampling/Venue-based sampling
◯ Snowball sampling
◯ Clinic/Facility-based sampling
◯ Outreach sampling
◯ Other

Specify (if other) ________________________

Longitudinal (Notes) __________________________________

RANDOMIZED CONTROLLED TRIAL

RCT (Sample size of control group) __________________________________

RCT (Sample size of intervention group) __________________________________

RCT (Intervention) __________________________________

RCT (Year of study start) __________________________________

RCT (Year of study end) __________________________________

RCT (Frequency of study visits)

- ◯ Daily
- ◯ Weekly
- ◯ Biweekly
- ◯ Monthly
- ◯ Every 3 months
- ◯ Every 6 months
- ◯ Annual

RCT (Recruitment method)

- ◯ Census
- ◯ Probability sampling
- ◯ Respondent driven sampling
- ◯ Time-Location Sampling/Venue-based sampling
- ◯ Snowball sampling
- ◯ Clinic/Facility-based sampling
- ◯ Outreach sampling
- ◯ Other

Specify (if other) __________________________________

RCT (Notes) __________________________________

SYSTEMATIC REVIEW/REVIEW

Systematic Review (Total sample size) __________________________________

Systematic Review (Number of studies included) __________________________________

Systematic Review (Year of study inclusion start, publication date) __________________________________

Systematic Review (Year of study inclusion end, publication date) __________________________________

Systematic Review (Meta-analysis conducted)

- ◯ Yes
- ◯ No

Systematic Review (Notes) __________________________________

Other study type, specify __________________________________

Region of interest

- ◯ Worldwide
- ◯ Eastern Africa
- ◯ Middle Africa
- ◯ Northern Africa
- ◯ Southern Africa
- ◯ Western Africa
- ◯ The Carribean
- ◯ Central America
- ◯ South America
- ◯ Northern America
- ◯ Asia
- ◯ Europe
- ◯ Middle East
- ◯ Oceania

Number of countries represented in article ______________________

Country 1:

◯ Afghanistan
◯ Albania
◯ Algeria
◯ Andorra
◯ Angola
◯ Antigua and Barbuda
◯ Argentina
◯ Armenia
◯ Aruba
◯ Australia
◯ Austria
◯ Azerbaijan
◯ Bahamas
◯ Bahrain
◯ Bangladesh
◯ Barbados
◯ Belarus
◯ Belgium
◯ Belize
◯ Benin
◯ Bhutan
◯ Bolivia
◯ Bosnia and Herzegovina
◯ Botswana
◯ Brazil
◯ Brunei
◯ Bulgaria
◯ Burkina Faso
◯ Burma
◯ Burundi
◯ Cambodia
◯ Cameroon
◯ Canada
◯ Cabo Verde
◯ Central African Republic
◯ Chad
◯ Chile
◯ China
◯ Colombia
◯ Comoros
◯ Republic of Congo
◯ Democratic Republic of Congo
◯ Costa Rica
◯ Cote d'Ivoire
◯ Croatia
◯ Cuba
◯ Curacao
◯ Cyprus
◯ Czechia
◯ Denmark
◯ Djibouti
◯ Dominica
◯ Dominican Republic
◯ Ecuador
◯ Egypt
◯ El Salvador
◯ Equatorial Guinea
◯ Eritrea
◯ Estonia
◯ Ethiopia
◯ Fiji
◯ Finland
◯ France
◯ Gabon
◯ The Gambia
◯ Georgia
◯ Germany
◯ Ghana
◯ Greece
◯ Grenada

◯ Guatemala
◯ Guinea
◯ Guinea-Bissau
◯ Guyana
◯ Haiti
◯ Holy See
◯ Honduras
◯ Hong Kong
◯ Hungary
◯ Iceland
◯ India
◯ Indonesia
◯ Iran
◯ Iraq
◯ Ireland
◯ Israel
◯ Italy
◯ Jamaica
◯ Japan
◯ Jordan
◯ Kazakhstan
◯ Kenya
◯ Kiribati
◯ North Korea
◯ South Korea
◯ Kosovo
◯ Kuwait
◯ Kyrgyzstan
◯ Laos
◯ Latvia
◯ Lebanon
◯ Lesotho
◯ Liberia
◯ Libya
◯ Liechtenstein
◯ Lithuania
◯ Luxembourg
◯ Macau
◯ Macedonia
◯ Madagascar
◯ Malawi
◯ Malaysia
◯ Maldives
◯ Mali
◯ Malta
◯ Marshall Islands
◯ Mauritania
◯ Mauritius
◯ Mexico
◯ Micronesia
◯ Moldova
◯ Monaco
◯ Mongolia
◯ Montenegro
◯ Morocco
◯ Mozambique
◯ Namibia
◯ Nauru
◯ Nepal
◯ Netherlands
◯ New Zealand
◯ Nicaragua
◯ Niger
◯ Nigeria
◯ North Korea
◯ Norway
◯ Oman
◯ Pakistan
◯ Palau
◯ Palestinian Territories
◯ Panama

◯ Papua New Guinea
◯ Paraguay
◯ Peru
◯ Philippines
◯ Poland
◯ Portugal
◯ Qatar
◯ Romania
◯ Russia
◯ Rwanda
◯ Saint Kitts and Nevis
◯ Saint Lucia
◯ Saint Vincent and the Grenadines
◯ Samoa
◯ San Marino
◯ Sao Tome and Principe
◯ Saudi Arabia
◯ Senegal
◯ Serbia
◯ Seychelles
◯ Sierra Leone
◯ Singapore
◯ Sint Maarten
◯ Slovakia
◯ Slovenia
◯ Solomon Islands
◯ Somalia
◯ South Africa
◯ South Korea
◯ South Sudan
◯ Spain
◯ Sri Lanka
◯ Sudan
◯ Suriname
◯ Swaziland
◯ Sweden
◯ Switzerland
◯ Syria
◯ Taiwan
◯ Tajikistan
◯ Tanzania
◯ Thailand
◯ Timor-Leste
◯ Togo
◯ Tonga
◯ Trinidad and Tobago
◯ Tunisia
◯ Turkey
◯ Turkmenistan
◯ Tuvalu
◯ Uganda
◯ Ukraine
◯ United Arab Emirates
◯ United Kingdom
◯ United States of America
◯ Uruguay
◯ Uzbekistan
◯ Vanuatu
◯ Venezuela
◯ Vietnam
◯ Yemen
◯ Zambia
◯ Zimbabwe

Country 2:

- ◯ Afghanistan
- ◯ Albania
- ◯ Algeria
- ◯ Andorra
- ◯ Angola
- ◯ Antigua and Barbuda
- ◯ Argentina
- ◯ Armenia
- ◯ Aruba
- ◯ Australia
- ◯ Austria
- ◯ Azerbaijan
- ◯ Bahamas
- ◯ Bahrain
- ◯ Bangladesh
- ◯ Barbados
- ◯ Belarus
- ◯ Belgium
- ◯ Belize
- ◯ Benin
- ◯ Bhutan
- ◯ Bolivia
- ◯ Bosnia and Herzegovina
- ◯ Botswana
- ◯ Brazil
- ◯ Brunei
- ◯ Bulgaria
- ◯ Burkina Faso
- ◯ Burma
- ◯ Burundi
- ◯ Cambodia
- ◯ Cameroon
- ◯ Canada
- ◯ Cabo Verde
- ◯ Central African Republic
- ◯ Chad
- ◯ Chile
- ◯ China
- ◯ Colombia
- ◯ Comoros
- ◯ Republic of Congo
- ◯ Democratic Republic of Congo
- ◯ Costa Rica
- ◯ Cote d'Ivoire
- ◯ Croatia
- ◯ Cuba
- ◯ Curacao
- ◯ Cyprus
- ◯ Czechia
- ◯ Denmark
- ◯ Djibouti
- ◯ Dominica
- ◯ Dominican Republic
- ◯ Ecuador
- ◯ Egypt
- ◯ El Salvador
- ◯ Equatorial Guinea
- ◯ Eritrea
- ◯ Estonia
- ◯ Ethiopia
- ◯ Fiji
- ◯ Finland
- ◯ France
- ◯ Gabon
- ◯ The Gambia
- ◯ Georgia
- ◯ Germany
- ◯ Ghana
- ◯ Greece
- ◯ Grenada

◯ Guatemala
◯ Guinea
◯ Guinea-Bissau
◯ Guyana
◯ Haiti
◯ Holy See
◯ Honduras
◯ Hong Kong
◯ Hungary
◯ Iceland
◯ India
◯ Indonesia
◯ Iran
◯ Iraq
◯ Ireland
◯ Israel
◯ Italy
◯ Jamaica
◯ Japan
◯ Jordan
◯ Kazakhstan
◯ Kenya
◯ Kiribati
◯ North Korea
◯ South Korea
◯ Kosovo
◯ Kuwait
◯ Kyrgyzstan
◯ Laos
◯ Latvia
◯ Lebanon
◯ Lesotho
◯ Liberia
◯ Libya
◯ Liechtenstein
◯ Lithuania
◯ Luxembourg
◯ Macau
◯ Macedonia
◯ Madagascar
◯ Malawi
◯ Malaysia
◯ Maldives
◯ Mali
◯ Malta
◯ Marshall Islands
◯ Mauritania
◯ Mauritius
◯ Mexico
◯ Micronesia
◯ Moldova
◯ Monaco
◯ Mongolia
◯ Montenegro
◯ Morocco
◯ Mozambique
◯ Namibia
◯ Nauru
◯ Nepal
◯ Netherlands
◯ New Zealand
◯ Nicaragua
◯ Niger
◯ Nigeria
◯ North Korea
◯ Norway
◯ Oman
◯ Pakistan
◯ Palau
◯ Palestinian Territories
◯ Panama

◯ Papua New Guinea
◯ Paraguay
◯ Peru
◯ Philippines
◯ Poland
◯ Portugal
◯ Qatar
◯ Romania
◯ Russia
◯ Rwanda
◯ Saint Kitts and Nevis
◯ Saint Lucia
◯ Saint Vincent and the Grenadines
◯ Samoa
◯ San Marino
◯ Sao Tome and Principe
◯ Saudi Arabia
◯ Senegal
◯ Serbia
◯ Seychelles
◯ Sierra Leone
◯ Singapore
◯ Sint Maarten
◯ Slovakia
◯ Slovenia
◯ Solomon Islands
◯ Somalia
◯ South Africa
◯ South Korea
◯ South Sudan
◯ Spain
◯ Sri Lanka
◯ Sudan
◯ Suriname
◯ Swaziland
◯ Sweden
◯ Switzerland
◯ Syria
◯ Taiwan
◯ Tajikistan
◯ Tanzania
◯ Thailand
◯ Timor-Leste
◯ Togo
◯ Tonga
◯ Trinidad and Tobago
◯ Tunisia
◯ Turkey
◯ Turkmenistan
◯ Tuvalu
◯ Uganda
◯ Ukraine
◯ United Arab Emirates
◯ United Kingdom
◯ United States of America
◯ Uruguay
◯ Uzbekistan
◯ Vanuatu
◯ Venezuela
◯ Vietnam
◯ Yemen
◯ Zambia
◯ Zimbabwe

Country 3:

◯ Afghanistan
◯ Albania
◯ Algeria
◯ Andorra
◯ Angola
◯ Antigua and Barbuda
◯ Argentina
◯ Armenia
◯ Aruba
◯ Australia
◯ Austria
◯ Azerbaijan
◯ Bahamas
◯ Bahrain
◯ Bangladesh
◯ Barbados
◯ Belarus
◯ Belgium
◯ Belize
◯ Benin
◯ Bhutan
◯ Bolivia
◯ Bosnia and Herzegovina
◯ Botswana
◯ Brazil
◯ Brunei
◯ Bulgaria
◯ Burkina Faso
◯ Burma
◯ Burundi
◯ Cambodia
◯ Cameroon
◯ Canada
◯ Cabo Verde
◯ Central African Republic
◯ Chad
◯ Chile
◯ China
◯ Colombia
◯ Comoros
◯ Republic of Congo
◯ Democratic Republic of Congo
◯ Costa Rica
◯ Cote d'Ivoire
◯ Croatia
◯ Cuba
◯ Curacao
◯ Cyprus
◯ Czechia
◯ Denmark
◯ Djibouti
◯ Dominica
◯ Dominican Republic
◯ Ecuador
◯ Egypt
◯ El Salvador
◯ Equatorial Guinea
◯ Eritrea
◯ Estonia
◯ Ethiopia
◯ Fiji
◯ Finland
◯ France
◯ Gabon
◯ The Gambia
◯ Georgia
◯ Germany
◯ Ghana
◯ Greece
◯ Grenada

◯ Guatemala
◯ Guinea
◯ Guinea-Bissau
◯ Guyana
◯ Haiti
◯ Holy See
◯ Honduras
◯ Hong Kong
◯ Hungary
◯ Iceland
◯ India
◯ Indonesia
◯ Iran
◯ Iraq
◯ Ireland
◯ Israel
◯ Italy
◯ Jamaica
◯ Japan
◯ Jordan
◯ Kazakhstan
◯ Kenya
◯ Kiribati
◯ North Korea
◯ South Korea
◯ Kosovo
◯ Kuwait
◯ Kyrgyzstan
◯ Laos
◯ Latvia
◯ Lebanon
◯ Lesotho
◯ Liberia
◯ Libya
◯ Liechtenstein
◯ Lithuania
◯ Luxembourg
◯ Macau
◯ Macedonia
◯ Madagascar
◯ Malawi
◯ Malaysia
◯ Maldives
◯ Mali
◯ Malta
◯ Marshall Islands
◯ Mauritania
◯ Mauritius
◯ Mexico
◯ Micronesia
◯ Moldova
◯ Monaco
◯ Mongolia
◯ Montenegro
◯ Morocco
◯ Mozambique
◯ Namibia
◯ Nauru
◯ Nepal
◯ Netherlands
◯ New Zealand
◯ Nicaragua
◯ Niger
◯ Nigeria
◯ North Korea
◯ Norway
◯ Oman
◯ Pakistan
◯ Palau
◯ Palestinian Territories
◯ Panama

◯ Papua New Guinea
◯ Paraguay
◯ Peru
◯ Philippines
◯ Poland
◯ Portugal
◯ Qatar
◯ Romania
◯ Russia
◯ Rwanda
◯ Saint Kitts and Nevis
◯ Saint Lucia
◯ Saint Vincent and the Grenadines
◯ Samoa
◯ San Marino
◯ Sao Tome and Principe
◯ Saudi Arabia
◯ Senegal
◯ Serbia
◯ Seychelles
◯ Sierra Leone
◯ Singapore
◯ Sint Maarten
◯ Slovakia
◯ Slovenia
◯ Solomon Islands
◯ Somalia
◯ South Africa
◯ South Korea
◯ South Sudan
◯ Spain
◯ Sri Lanka
◯ Sudan
◯ Suriname
◯ Swaziland
◯ Sweden
◯ Switzerland
◯ Syria
◯ Taiwan
◯ Tajikistan
◯ Tanzania
◯ Thailand
◯ Timor-Leste
◯ Togo
◯ Tonga
◯ Trinidad and Tobago
◯ Tunisia
◯ Turkey
◯ Turkmenistan
◯ Tuvalu
◯ Uganda
◯ Ukraine
◯ United Arab Emirates
◯ United Kingdom
◯ United States of America
◯ Uruguay
◯ Uzbekistan
◯ Vanuatu
◯ Venezuela
◯ Vietnam
◯ Yemen
◯ Zambia
◯ Zimbabwe

Country 4:

◯ Afghanistan
◯ Albania
◯ Algeria
◯ Andorra
◯ Angola
◯ Antigua and Barbuda
◯ Argentina
◯ Armenia
◯ Aruba
◯ Australia
◯ Austria
◯ Azerbaijan
◯ Bahamas
◯ Bahrain
◯ Bangladesh
◯ Barbados
◯ Belarus
◯ Belgium
◯ Belize
◯ Benin
◯ Bhutan
◯ Bolivia
◯ Bosnia and Herzegovina
◯ Botswana
◯ Brazil
◯ Brunei
◯ Bulgaria
◯ Burkina Faso
◯ Burma
◯ Burundi
◯ Cambodia
◯ Cameroon
◯ Canada
◯ Cabo Verde
◯ Central African Republic
◯ Chad
◯ Chile
◯ China
◯ Colombia
◯ Comoros
◯ Republic of Congo
◯ Democratic Republic of Congo
◯ Costa Rica
◯ Cote d'Ivoire
◯ Croatia
◯ Cuba
◯ Curacao
◯ Cyprus
◯ Czechia
◯ Denmark
◯ Djibouti
◯ Dominica
◯ Dominican Republic
◯ Ecuador
◯ Egypt
◯ El Salvador
◯ Equatorial Guinea
◯ Eritrea
◯ Estonia
◯ Ethiopia
◯ Fiji
◯ Finland
◯ France
◯ Gabon
◯ The Gambia
◯ Georgia
◯ Germany
◯ Ghana
◯ Greece
◯ Grenada

◯ Guatemala
◯ Guinea
◯ Guinea-Bissau
◯ Guyana
◯ Haiti
◯ Holy See
◯ Honduras
◯ Hong Kong
◯ Hungary
◯ Iceland
◯ India
◯ Indonesia
◯ Iran
◯ Iraq
◯ Ireland
◯ Israel
◯ Italy
◯ Jamaica
◯ Japan
◯ Jordan
◯ Kazakhstan
◯ Kenya
◯ Kiribati
◯ North Korea
◯ South Korea
◯ Kosovo
◯ Kuwait
◯ Kyrgyzstan
◯ Laos
◯ Latvia
◯ Lebanon
◯ Lesotho
◯ Liberia
◯ Libya
◯ Liechtenstein
◯ Lithuania
◯ Luxembourg
◯ Macau
◯ Macedonia
◯ Madagascar
◯ Malawi
◯ Malaysia
◯ Maldives
◯ Mali
◯ Malta
◯ Marshall Islands
◯ Mauritania
◯ Mauritius
◯ Mexico
◯ Micronesia
◯ Moldova
◯ Monaco
◯ Mongolia
◯ Montenegro
◯ Morocco
◯ Mozambique
◯ Namibia
◯ Nauru
◯ Nepal
◯ Netherlands
◯ New Zealand
◯ Nicaragua
◯ Niger
◯ Nigeria
◯ North Korea
◯ Norway
◯ Oman
◯ Pakistan
◯ Palau
◯ Palestinian Territories
◯ Panama

◯ Papua New Guinea
◯ Paraguay
◯ Peru
◯ Philippines
◯ Poland
◯ Portugal
◯ Qatar
◯ Romania
◯ Russia
◯ Rwanda
◯ Saint Kitts and Nevis
◯ Saint Lucia
◯ Saint Vincent and the Grenadines
◯ Samoa
◯ San Marino
◯ Sao Tome and Principe
◯ Saudi Arabia
◯ Senegal
◯ Serbia
◯ Seychelles
◯ Sierra Leone
◯ Singapore
◯ Sint Maarten
◯ Slovakia
◯ Slovenia
◯ Solomon Islands
◯ Somalia
◯ South Africa
◯ South Korea
◯ South Sudan
◯ Spain
◯ Sri Lanka
◯ Sudan
◯ Suriname
◯ Swaziland
◯ Sweden
◯ Switzerland
◯ Syria
◯ Taiwan
◯ Tajikistan
◯ Tanzania
◯ Thailand
◯ Timor-Leste
◯ Togo
◯ Tonga
◯ Trinidad and Tobago
◯ Tunisia
◯ Turkey
◯ Turkmenistan
◯ Tuvalu
◯ Uganda
◯ Ukraine
◯ United Arab Emirates
◯ United Kingdom
◯ United States of America
◯ Uruguay
◯ Uzbekistan
◯ Vanuatu
◯ Venezuela
◯ Vietnam
◯ Yemen
◯ Zambia
◯ Zimbabwe

Country 5:

◯ Afghanistan
◯ Albania
◯ Algeria
◯ Andorra
◯ Angola
◯ Antigua and Barbuda
◯ Argentina
◯ Armenia
◯ Aruba
◯ Australia
◯ Austria
◯ Azerbaijan
◯ Bahamas
◯ Bahrain
◯ Bangladesh
◯ Barbados
◯ Belarus
◯ Belgium
◯ Belize
◯ Benin
◯ Bhutan
◯ Bolivia
◯ Bosnia and Herzegovina
◯ Botswana
◯ Brazil
◯ Brunei
◯ Bulgaria
◯ Burkina Faso
◯ Burma
◯ Burundi
◯ Cambodia
◯ Cameroon
◯ Canada
◯ Cabo Verde
◯ Central African Republic
◯ Chad
◯ Chile
◯ China
◯ Colombia
◯ Comoros
◯ Republic of Congo
◯ Democratic Republic of Congo
◯ Costa Rica
◯ Cote d'Ivoire
◯ Croatia
◯ Cuba
◯ Curacao
◯ Cyprus
◯ Czechia
◯ Denmark
◯ Djibouti
◯ Dominica
◯ Dominican Republic
◯ Ecuador
◯ Egypt
◯ El Salvador
◯ Equatorial Guinea
◯ Eritrea
◯ Estonia
◯ Ethiopia
◯ Fiji
◯ Finland
◯ France
◯ Gabon
◯ The Gambia
◯ Georgia
◯ Germany
◯ Ghana
◯ Greece
◯ Grenada

◯ Guatemala
◯ Guinea
◯ Guinea-Bissau
◯ Guyana
◯ Haiti
◯ Holy See
◯ Honduras
◯ Hong Kong
◯ Hungary
◯ Iceland
◯ India
◯ Indonesia
◯ Iran
◯ Iraq
◯ Ireland
◯ Israel
◯ Italy
◯ Jamaica
◯ Japan
◯ Jordan
◯ Kazakhstan
◯ Kenya
◯ Kiribati
◯ North Korea
◯ South Korea
◯ Kosovo
◯ Kuwait
◯ Kyrgyzstan
◯ Laos
◯ Latvia
◯ Lebanon
◯ Lesotho
◯ Liberia
◯ Libya
◯ Liechtenstein
◯ Lithuania
◯ Luxembourg
◯ Macau
◯ Macedonia
◯ Madagascar
◯ Malawi
◯ Malaysia
◯ Maldives
◯ Mali
◯ Malta
◯ Marshall Islands
◯ Mauritania
◯ Mauritius
◯ Mexico
◯ Micronesia
◯ Moldova
◯ Monaco
◯ Mongolia
◯ Montenegro
◯ Morocco
◯ Mozambique
◯ Namibia
◯ Nauru
◯ Nepal
◯ Netherlands
◯ New Zealand
◯ Nicaragua
◯ Niger
◯ Nigeria
◯ North Korea
◯ Norway
◯ Oman
◯ Pakistan
◯ Palau
◯ Palestinian Territories
◯ Panama

◯ Papua New Guinea
◯ Paraguay
◯ Peru
◯ Philippines
◯ Poland
◯ Portugal
◯ Qatar
◯ Romania
◯ Russia
◯ Rwanda
◯ Saint Kitts and Nevis
◯ Saint Lucia
◯ Saint Vincent and the Grenadines
◯ Samoa
◯ San Marino
◯ Sao Tome and Principe
◯ Saudi Arabia
◯ Senegal
◯ Serbia
◯ Seychelles
◯ Sierra Leone
◯ Singapore
◯ Sint Maarten
◯ Slovakia
◯ Slovenia
◯ Solomon Islands
◯ Somalia
◯ South Africa
◯ South Korea
◯ South Sudan
◯ Spain
◯ Sri Lanka
◯ Sudan
◯ Suriname
◯ Swaziland
◯ Sweden
◯ Switzerland
◯ Syria
◯ Taiwan
◯ Tajikistan
◯ Tanzania
◯ Thailand
◯ Timor-Leste
◯ Togo
◯ Tonga
◯ Trinidad and Tobago
◯ Tunisia
◯ Turkey
◯ Turkmenistan
◯ Tuvalu
◯ Uganda
◯ Ukraine
◯ United Arab Emirates
◯ United Kingdom
◯ United States of America
◯ Uruguay
◯ Uzbekistan
◯ Vanuatu
◯ Venezuela
◯ Vietnam
◯ Yemen
◯ Zambia
◯ Zimbabwe

Country 6:

◯ Afghanistan
◯ Albania
◯ Algeria
◯ Andorra
◯ Angola
◯ Antigua and Barbuda
◯ Argentina
◯ Armenia
◯ Aruba
◯ Australia
◯ Austria
◯ Azerbaijan
◯ Bahamas
◯ Bahrain
◯ Bangladesh
◯ Barbados
◯ Belarus
◯ Belgium
◯ Belize
◯ Benin
◯ Bhutan
◯ Bolivia
◯ Bosnia and Herzegovina
◯ Botswana
◯ Brazil
◯ Brunei
◯ Bulgaria
◯ Burkina Faso
◯ Burma
◯ Burundi
◯ Cambodia
◯ Cameroon
◯ Canada
◯ Cabo Verde
◯ Central African Republic
◯ Chad
◯ Chile
◯ China
◯ Colombia
◯ Comoros
◯ Republic of Congo
◯ Democratic Republic of Congo
◯ Costa Rica
◯ Cote d'Ivoire
◯ Croatia
◯ Cuba
◯ Curacao
◯ Cyprus
◯ Czechia
◯ Denmark
◯ Djibouti
◯ Dominica
◯ Dominican Republic
◯ Ecuador
◯ Egypt
◯ El Salvador
◯ Equatorial Guinea
◯ Eritrea
◯ Estonia
◯ Ethiopia
◯ Fiji
◯ Finland
◯ France
◯ Gabon
◯ The Gambia
◯ Georgia
◯ Germany
◯ Ghana
◯ Greece
◯ Grenada

◯ Guatemala
◯ Guinea
◯ Guinea-Bissau
◯ Guyana
◯ Haiti
◯ Holy See
◯ Honduras
◯ Hong Kong
◯ Hungary
◯ Iceland
◯ India
◯ Indonesia
◯ Iran
◯ Iraq
◯ Ireland
◯ Israel
◯ Italy
◯ Jamaica
◯ Japan
◯ Jordan
◯ Kazakhstan
◯ Kenya
◯ Kiribati
◯ North Korea
◯ South Korea
◯ Kosovo
◯ Kuwait
◯ Kyrgyzstan
◯ Laos
◯ Latvia
◯ Lebanon
◯ Lesotho
◯ Liberia
◯ Libya
◯ Liechtenstein
◯ Lithuania
◯ Luxembourg
◯ Macau
◯ Macedonia
◯ Madagascar
◯ Malawi
◯ Malaysia
◯ Maldives
◯ Mali
◯ Malta
◯ Marshall Islands
◯ Mauritania
◯ Mauritius
◯ Mexico
◯ Micronesia
◯ Moldova
◯ Monaco
◯ Mongolia
◯ Montenegro
◯ Morocco
◯ Mozambique
◯ Namibia
◯ Nauru
◯ Nepal
◯ Netherlands
◯ New Zealand
◯ Nicaragua
◯ Niger
◯ Nigeria
◯ North Korea
◯ Norway
◯ Oman
◯ Pakistan
◯ Palau
◯ Palestinian Territories
◯ Panama

◯ Papua New Guinea
◯ Paraguay
◯ Peru
◯ Philippines
◯ Poland
◯ Portugal
◯ Qatar
◯ Romania
◯ Russia
◯ Rwanda
◯ Saint Kitts and Nevis
◯ Saint Lucia
◯ Saint Vincent and the Grenadines
◯ Samoa
◯ San Marino
◯ Sao Tome and Principe
◯ Saudi Arabia
◯ Senegal
◯ Serbia
◯ Seychelles
◯ Sierra Leone
◯ Singapore
◯ Sint Maarten
◯ Slovakia
◯ Slovenia
◯ Solomon Islands
◯ Somalia
◯ South Africa
◯ South Korea
◯ South Sudan
◯ Spain
◯ Sri Lanka
◯ Sudan
◯ Suriname
◯ Swaziland
◯ Sweden
◯ Switzerland
◯ Syria
◯ Taiwan
◯ Tajikistan
◯ Tanzania
◯ Thailand
◯ Timor-Leste
◯ Togo
◯ Tonga
◯ Trinidad and Tobago
◯ Tunisia
◯ Turkey
◯ Turkmenistan
◯ Tuvalu
◯ Uganda
◯ Ukraine
◯ United Arab Emirates
◯ United Kingdom
◯ United States of America
◯ Uruguay
◯ Uzbekistan
◯ Vanuatu
◯ Venezuela
◯ Vietnam
◯ Yemen
◯ Zambia
◯ Zimbabwe

Country 7:

◯ Afghanistan
◯ Albania
◯ Algeria
◯ Andorra
◯ Angola
◯ Antigua and Barbuda
◯ Argentina
◯ Armenia
◯ Aruba
◯ Australia
◯ Austria
◯ Azerbaijan
◯ Bahamas
◯ Bahrain
◯ Bangladesh
◯ Barbados
◯ Belarus
◯ Belgium
◯ Belize
◯ Benin
◯ Bhutan
◯ Bolivia
◯ Bosnia and Herzegovina
◯ Botswana
◯ Brazil
◯ Brunei
◯ Bulgaria
◯ Burkina Faso
◯ Burma
◯ Burundi
◯ Cambodia
◯ Cameroon
◯ Canada
◯ Cabo Verde
◯ Central African Republic
◯ Chad
◯ Chile
◯ China
◯ Colombia
◯ Comoros
◯ Republic of Congo
◯ Democratic Republic of Congo
◯ Costa Rica
◯ Cote d'Ivoire
◯ Croatia
◯ Cuba
◯ Curacao
◯ Cyprus
◯ Czechia
◯ Denmark
◯ Djibouti
◯ Dominica
◯ Dominican Republic
◯ Ecuador
◯ Egypt
◯ El Salvador
◯ Equatorial Guinea
◯ Eritrea
◯ Estonia
◯ Ethiopia
◯ Fiji
◯ Finland
◯ France
◯ Gabon
◯ The Gambia
◯ Georgia
◯ Germany
◯ Ghana
◯ Greece
◯ Grenada

◯ Guatemala
◯ Guinea
◯ Guinea-Bissau
◯ Guyana
◯ Haiti
◯ Holy See
◯ Honduras
◯ Hong Kong
◯ Hungary
◯ Iceland
◯ India
◯ Indonesia
◯ Iran
◯ Iraq
◯ Ireland
◯ Israel
◯ Italy
◯ Jamaica
◯ Japan
◯ Jordan
◯ Kazakhstan
◯ Kenya
◯ Kiribati
◯ North Korea
◯ South Korea
◯ Kosovo
◯ Kuwait
◯ Kyrgyzstan
◯ Laos
◯ Latvia
◯ Lebanon
◯ Lesotho
◯ Liberia
◯ Libya
◯ Liechtenstein
◯ Lithuania
◯ Luxembourg
◯ Macau
◯ Macedonia
◯ Madagascar
◯ Malawi
◯ Malaysia
◯ Maldives
◯ Mali
◯ Malta
◯ Marshall Islands
◯ Mauritania
◯ Mauritius
◯ Mexico
◯ Micronesia
◯ Moldova
◯ Monaco
◯ Mongolia
◯ Montenegro
◯ Morocco
◯ Mozambique
◯ Namibia
◯ Nauru
◯ Nepal
◯ Netherlands
◯ New Zealand
◯ Nicaragua
◯ Niger
◯ Nigeria
◯ North Korea
◯ Norway
◯ Oman
◯ Pakistan
◯ Palau
◯ Palestinian Territories
◯ Panama

◯ Papua New Guinea
◯ Paraguay
◯ Peru
◯ Philippines
◯ Poland
◯ Portugal
◯ Qatar
◯ Romania
◯ Russia
◯ Rwanda
◯ Saint Kitts and Nevis
◯ Saint Lucia
◯ Saint Vincent and the Grenadines
◯ Samoa
◯ San Marino
◯ Sao Tome and Principe
◯ Saudi Arabia
◯ Senegal
◯ Serbia
◯ Seychelles
◯ Sierra Leone
◯ Singapore
◯ Sint Maarten
◯ Slovakia
◯ Slovenia
◯ Solomon Islands
◯ Somalia
◯ South Africa
◯ South Korea
◯ South Sudan
◯ Spain
◯ Sri Lanka
◯ Sudan
◯ Suriname
◯ Swaziland
◯ Sweden
◯ Switzerland
◯ Syria
◯ Taiwan
◯ Tajikistan
◯ Tanzania
◯ Thailand
◯ Timor-Leste
◯ Togo
◯ Tonga
◯ Trinidad and Tobago
◯ Tunisia
◯ Turkey
◯ Turkmenistan
◯ Tuvalu
◯ Uganda
◯ Ukraine
◯ United Arab Emirates
◯ United Kingdom
◯ United States of America
◯ Uruguay
◯ Uzbekistan
◯ Vanuatu
◯ Venezuela
◯ Vietnam
◯ Yemen
◯ Zambia
◯ Zimbabwe

Country 8:

◯ Afghanistan
◯ Albania
◯ Algeria
◯ Andorra
◯ Angola
◯ Antigua and Barbuda
◯ Argentina
◯ Armenia
◯ Aruba
◯ Australia
◯ Austria
◯ Azerbaijan
◯ Bahamas
◯ Bahrain
◯ Bangladesh
◯ Barbados
◯ Belarus
◯ Belgium
◯ Belize
◯ Benin
◯ Bhutan
◯ Bolivia
◯ Bosnia and Herzegovina
◯ Botswana
◯ Brazil
◯ Brunei
◯ Bulgaria
◯ Burkina Faso
◯ Burma
◯ Burundi
◯ Cambodia
◯ Cameroon
◯ Canada
◯ Cabo Verde
◯ Central African Republic
◯ Chad
◯ Chile
◯ China
◯ Colombia
◯ Comoros
◯ Republic of Congo
◯ Democratic Republic of Congo
◯ Costa Rica
◯ Cote d'Ivoire
◯ Croatia
◯ Cuba
◯ Curacao
◯ Cyprus
◯ Czechia
◯ Denmark
◯ Djibouti
◯ Dominica
◯ Dominican Republic
◯ Ecuador
◯ Egypt
◯ El Salvador
◯ Equatorial Guinea
◯ Eritrea
◯ Estonia
◯ Ethiopia
◯ Fiji
◯ Finland
◯ France
◯ Gabon
◯ The Gambia
◯ Georgia
◯ Germany
◯ Ghana
◯ Greece
◯ Grenada

◯ Guatemala
◯ Guinea
◯ Guinea-Bissau
◯ Guyana
◯ Haiti
◯ Holy See
◯ Honduras
◯ Hong Kong
◯ Hungary
◯ Iceland
◯ India
◯ Indonesia
◯ Iran
◯ Iraq
◯ Ireland
◯ Israel
◯ Italy
◯ Jamaica
◯ Japan
◯ Jordan
◯ Kazakhstan
◯ Kenya
◯ Kiribati
◯ North Korea
◯ South Korea
◯ Kosovo
◯ Kuwait
◯ Kyrgyzstan
◯ Laos
◯ Latvia
◯ Lebanon
◯ Lesotho
◯ Liberia
◯ Libya
◯ Liechtenstein
◯ Lithuania
◯ Luxembourg
◯ Macau
◯ Macedonia
◯ Madagascar
◯ Malawi
◯ Malaysia
◯ Maldives
◯ Mali
◯ Malta
◯ Marshall Islands
◯ Mauritania
◯ Mauritius
◯ Mexico
◯ Micronesia
◯ Moldova
◯ Monaco
◯ Mongolia
◯ Montenegro
◯ Morocco
◯ Mozambique
◯ Namibia
◯ Nauru
◯ Nepal
◯ Netherlands
◯ New Zealand
◯ Nicaragua
◯ Niger
◯ Nigeria
◯ North Korea
◯ Norway
◯ Oman
◯ Pakistan
◯ Palau
◯ Palestinian Territories
◯ Panama

◯ Papua New Guinea
◯ Paraguay
◯ Peru
◯ Philippines
◯ Poland
◯ Portugal
◯ Qatar
◯ Romania
◯ Russia
◯ Rwanda
◯ Saint Kitts and Nevis
◯ Saint Lucia
◯ Saint Vincent and the Grenadines
◯ Samoa
◯ San Marino
◯ Sao Tome and Principe
◯ Saudi Arabia
◯ Senegal
◯ Serbia
◯ Seychelles
◯ Sierra Leone
◯ Singapore
◯ Sint Maarten
◯ Slovakia
◯ Slovenia
◯ Solomon Islands
◯ Somalia
◯ South Africa
◯ South Korea
◯ South Sudan
◯ Spain
◯ Sri Lanka
◯ Sudan
◯ Suriname
◯ Swaziland
◯ Sweden
◯ Switzerland
◯ Syria
◯ Taiwan
◯ Tajikistan
◯ Tanzania
◯ Thailand
◯ Timor-Leste
◯ Togo
◯ Tonga
◯ Trinidad and Tobago
◯ Tunisia
◯ Turkey
◯ Turkmenistan
◯ Tuvalu
◯ Uganda
◯ Ukraine
◯ United Arab Emirates
◯ United Kingdom
◯ United States of America
◯ Uruguay
◯ Uzbekistan
◯ Vanuatu
◯ Venezuela
◯ Vietnam
◯ Yemen
◯ Zambia
◯ Zimbabwe

Country 9:

◯ Afghanistan
◯ Albania
◯ Algeria
◯ Andorra
◯ Angola
◯ Antigua and Barbuda
◯ Argentina
◯ Armenia
◯ Aruba
◯ Australia
◯ Austria
◯ Azerbaijan
◯ Bahamas
◯ Bahrain
◯ Bangladesh
◯ Barbados
◯ Belarus
◯ Belgium
◯ Belize
◯ Benin
◯ Bhutan
◯ Bolivia
◯ Bosnia and Herzegovina
◯ Botswana
◯ Brazil
◯ Brunei
◯ Bulgaria
◯ Burkina Faso
◯ Burma
◯ Burundi
◯ Cambodia
◯ Cameroon
◯ Canada
◯ Cabo Verde
◯ Central African Republic
◯ Chad
◯ Chile
◯ China
◯ Colombia
◯ Comoros
◯ Republic of Congo
◯ Democratic Republic of Congo
◯ Costa Rica
◯ Cote d'Ivoire
◯ Croatia
◯ Cuba
◯ Curacao
◯ Cyprus
◯ Czechia
◯ Denmark
◯ Djibouti
◯ Dominica
◯ Dominican Republic
◯ Ecuador
◯ Egypt
◯ El Salvador
◯ Equatorial Guinea
◯ Eritrea
◯ Estonia
◯ Ethiopia
◯ Fiji
◯ Finland
◯ France
◯ Gabon
◯ The Gambia
◯ Georgia
◯ Germany
◯ Ghana
◯ Greece
◯ Grenada

◯ Guatemala
◯ Guinea
◯ Guinea-Bissau
◯ Guyana
◯ Haiti
◯ Holy See
◯ Honduras
◯ Hong Kong
◯ Hungary
◯ Iceland
◯ India
◯ Indonesia
◯ Iran
◯ Iraq
◯ Ireland
◯ Israel
◯ Italy
◯ Jamaica
◯ Japan
◯ Jordan
◯ Kazakhstan
◯ Kenya
◯ Kiribati
◯ North Korea
◯ South Korea
◯ Kosovo
◯ Kuwait
◯ Kyrgyzstan
◯ Laos
◯ Latvia
◯ Lebanon
◯ Lesotho
◯ Liberia
◯ Libya
◯ Liechtenstein
◯ Lithuania
◯ Luxembourg
◯ Macau
◯ Macedonia
◯ Madagascar
◯ Malawi
◯ Malaysia
◯ Maldives
◯ Mali
◯ Malta
◯ Marshall Islands
◯ Mauritania
◯ Mauritius
◯ Mexico
◯ Micronesia
◯ Moldova
◯ Monaco
◯ Mongolia
◯ Montenegro
◯ Morocco
◯ Mozambique
◯ Namibia
◯ Nauru
◯ Nepal
◯ Netherlands
◯ New Zealand
◯ Nicaragua
◯ Niger
◯ Nigeria
◯ North Korea
◯ Norway
◯ Oman
◯ Pakistan
◯ Palau
◯ Palestinian Territories
◯ Panama

◯ Papua New Guinea
◯ Paraguay
◯ Peru
◯ Philippines
◯ Poland
◯ Portugal
◯ Qatar
◯ Romania
◯ Russia
◯ Rwanda
◯ Saint Kitts and Nevis
◯ Saint Lucia
◯ Saint Vincent and the Grenadines
◯ Samoa
◯ San Marino
◯ Sao Tome and Principe
◯ Saudi Arabia
◯ Senegal
◯ Serbia
◯ Seychelles
◯ Sierra Leone
◯ Singapore
◯ Sint Maarten
◯ Slovakia
◯ Slovenia
◯ Solomon Islands
◯ Somalia
◯ South Africa
◯ South Korea
◯ South Sudan
◯ Spain
◯ Sri Lanka
◯ Sudan
◯ Suriname
◯ Swaziland
◯ Sweden
◯ Switzerland
◯ Syria
◯ Taiwan
◯ Tajikistan
◯ Tanzania
◯ Thailand
◯ Timor-Leste
◯ Togo
◯ Tonga
◯ Trinidad and Tobago
◯ Tunisia
◯ Turkey
◯ Turkmenistan
◯ Tuvalu
◯ Uganda
◯ Ukraine
◯ United Arab Emirates
◯ United Kingdom
◯ United States of America
◯ Uruguay
◯ Uzbekistan
◯ Vanuatu
◯ Venezuela
◯ Vietnam
◯ Yemen
◯ Zambia
◯ Zimbabwe

Country 10:

◯ Afghanistan
◯ Albania
◯ Algeria
◯ Andorra
◯ Angola
◯ Antigua and Barbuda
◯ Argentina
◯ Armenia
◯ Aruba
◯ Australia
◯ Austria
◯ Azerbaijan
◯ Bahamas
◯ Bahrain
◯ Bangladesh
◯ Barbados
◯ Belarus
◯ Belgium
◯ Belize
◯ Benin
◯ Bhutan
◯ Bolivia
◯ Bosnia and Herzegovina
◯ Botswana
◯ Brazil
◯ Brunei
◯ Bulgaria
◯ Burkina Faso
◯ Burma
◯ Burundi
◯ Cambodia
◯ Cameroon
◯ Canada
◯ Cabo Verde
◯ Central African Republic
◯ Chad
◯ Chile
◯ China
◯ Colombia
◯ Comoros
◯ Republic of Congo
◯ Democratic Republic of Congo
◯ Costa Rica
◯ Cote d'Ivoire
◯ Croatia
◯ Cuba
◯ Curacao
◯ Cyprus
◯ Czechia
◯ Denmark
◯ Djibouti
◯ Dominica
◯ Dominican Republic
◯ Ecuador
◯ Egypt
◯ El Salvador
◯ Equatorial Guinea
◯ Eritrea
◯ Estonia
◯ Ethiopia
◯ Fiji
◯ Finland
◯ France
◯ Gabon
◯ The Gambia
◯ Georgia
◯ Germany
◯ Ghana
◯ Greece
◯ Grenada

◯ Guatemala
◯ Guinea
◯ Guinea-Bissau
◯ Guyana
◯ Haiti
◯ Holy See
◯ Honduras
◯ Hong Kong
◯ Hungary
◯ Iceland
◯ India
◯ Indonesia
◯ Iran
◯ Iraq
◯ Ireland
◯ Israel
◯ Italy
◯ Jamaica
◯ Japan
◯ Jordan
◯ Kazakhstan
◯ Kenya
◯ Kiribati
◯ North Korea
◯ South Korea
◯ Kosovo
◯ Kuwait
◯ Kyrgyzstan
◯ Laos
◯ Latvia
◯ Lebanon
◯ Lesotho
◯ Liberia
◯ Libya
◯ Liechtenstein
◯ Lithuania
◯ Luxembourg
◯ Macau
◯ Macedonia
◯ Madagascar
◯ Malawi
◯ Malaysia
◯ Maldives
◯ Mali
◯ Malta
◯ Marshall Islands
◯ Mauritania
◯ Mauritius
◯ Mexico
◯ Micronesia
◯ Moldova
◯ Monaco
◯ Mongolia
◯ Montenegro
◯ Morocco
◯ Mozambique
◯ Namibia
◯ Nauru
◯ Nepal
◯ Netherlands
◯ New Zealand
◯ Nicaragua
◯ Niger
◯ Nigeria
◯ North Korea
◯ Norway
◯ Oman
◯ Pakistan
◯ Palau
◯ Palestinian Territories
◯ Panama

◯ Papua New Guinea
◯ Paraguay
◯ Peru
◯ Philippines
◯ Poland
◯ Portugal
◯ Qatar
◯ Romania
◯ Russia
◯ Rwanda
◯ Saint Kitts and Nevis
◯ Saint Lucia
◯ Saint Vincent and the Grenadines
◯ Samoa
◯ San Marino
◯ Sao Tome and Principe
◯ Saudi Arabia
◯ Senegal
◯ Serbia
◯ Seychelles
◯ Sierra Leone
◯ Singapore
◯ Sint Maarten
◯ Slovakia
◯ Slovenia
◯ Solomon Islands
◯ Somalia
◯ South Africa
◯ South Korea
◯ South Sudan
◯ Spain
◯ Sri Lanka
◯ Sudan
◯ Suriname
◯ Swaziland
◯ Sweden
◯ Switzerland
◯ Syria
◯ Taiwan
◯ Tajikistan
◯ Tanzania
◯ Thailand
◯ Timor-Leste
◯ Togo
◯ Tonga
◯ Trinidad and Tobago
◯ Tunisia
◯ Turkey
◯ Turkmenistan
◯ Tuvalu
◯ Uganda
◯ Ukraine
◯ United Arab Emirates
◯ United Kingdom
◯ United States of America
◯ Uruguay
◯ Uzbekistan
◯ Vanuatu
◯ Venezuela
◯ Vietnam
◯ Yemen
◯ Zambia
◯ Zimbabwe

Number of populations of interest ________________________________

Population of interest 1:
- ◯ Female Sex Workers
- ◯ Gay men and other men who have sex with men
- ◯ People who use drugs
- ◯ Transgender people
- ◯ Incarcerated populations

Population of interest 2:
- ◯ Female Sex Workers
- ◯ Gay men and other men who have sex with men
- ◯ People who use drugs
- ◯ Transgender people
- ◯ Incarcerated populations

Population of interest 3:
- ◯ Female Sex Workers
- ◯ Gay men and other men who have sex with men
- ◯ People who use drugs
- ◯ Transgender people
- ◯ Incarcerated populations

Population of interest 4:
- ◯ Female Sex Workers
- ◯ Gay men and other men who have sex with men
- ◯ People who use drugs
- ◯ Transgender people
- ◯ Incarcerated populations

Population of interest 5:
- ◯ Female Sex Workers
- ◯ Gay men and other men who have sex with men
- ◯ People who use drugs
- ◯ Transgender people
- ◯ Incarcerated populations

FEMALE SEX WORKERS

Sample size of FSW ________________________

BURDEN OF HIV (FSW)

Prevalence of HIV in sample (count) ________________________

(FSW)

Prevalence, lower bound of CI - reported ________________________

(FSW)

Prevalence, upper bound of CI - reported ________________________

(FSW)

Prevalence, lower bound of CI - calculated ________________________

(FSW)

Prevalence, upper bound of CI - calculated ________________________

(FSW)

Incidence of HIV in sample (count) ________________________

(FSW)

Incidence, lower bound of CI - reported ________________________

(FSW)

Incidence, upper bound of CI - reported ________________________

(FSW)

Incidence, lower bound of CI - calculated ________________________

(FSW)

Incidence, upper bound of CI - calculated ________________________

(FSW)

Notes, burden of disease

____

(FSW)

HIV PREVENTION CASCADE

Prevention cascade denominator (number of HIV negative) ________________________

Number of HIV negative individuals tested for HIV ________________________

(FSW)

Number of HIV negative individuals tested for HIV, lower bound of CI - calculated ________________________

(FSW)

Number of HIV negative individuals tested for HIV, upper bound of CI - calculated ________________________

(FSW)

Number tested negative __________________________________

(FSW)

Number tested negative, lower bound of CI - calculated __________________________________

(FSW)

Number tested negative, upper bound of CI - calculated __________________________________

(FSW)

Number with knowledge of HIV prevention __________________________________

(FSW)

Number with knowledge of HIV prevention, lower bound of CI - calculated __________________________________

(FSW)

Number with knowledge of HIV prevention, upper bound of CI - calculated __________________________________

(FSW)

Number who report condom availability __________________________________

(FSW)

Number who report condom availability, lower bound of CI - calculated __________________________________

(FSW)

Number who report condom availability, upper bound of CI - calculated __________________________________

(FSW)

Number who report PrEP availability __________________________________

(FSW)

Number who report PrEP availability, lower bound of CI - calculated __________________________________

(FSW)

Number who report PrEP availability, upper bound of CI - calculated ________________________________

(FSW)

Notes, prevention cascade

____

(FSW)

HIV TREATMENT CASCADE

Treatment cascade denominator ________________________________

Number tested (ever) ________________________________

(FSW)

Number tested (ever), lower bound of CI - calculated ________________________________

(FSW)

Number tested (ever), upper bound of CI - calculated ________________________________

(FSW)

Number tested in last 12 months ________________________________

(FSW)

Number tested in last 12 months, lower bound of CI - calculated ________________________________

(FSW)

Number tested in last 12 months, upper bound of CI - calculated ________________________________

(FSW)

Number previously diagnosed ________________________________

(FSW)

Number previously diagnosed, lower bound of CI - calculated __________________________________

(FSW)

Number previously diagnosed, upper bound of CI - calculated __________________________________

(FSW)

Number linked to HIV care __________________________________

(FSW)

Number linked to HIV care, lower bound of CI - calculated __________________________________

(FSW)

Number linked to HIV care, upper bound of CI - calculated __________________________________

(FSW)

Number retained in care __________________________________

(FSW)

Number retained in care, lower bound of CI - calculated __________________________________

(FSW)

Number retained in care, upper bound of CI - calculated __________________________________

(FSW)

Number on treatment __________________________________

(FSW)

Number on treatment, lower bound of CI - calculated __________________________________

(FSW)

Number on treatment, upper bound of CI - calculated ________________________

(FSW)

Number adherent/undetectable ________________________

(FSW)

Number adherent/undetectable, lower bound of CI - calculated ________________________

(FSW)

Number adherent/undetectable, upper bound of CI - calculated ________________________

(FSW)

Notes, treatment cascade

____

(FSW)

POPULATION SIZE ESTIMATES

Size estimate 1 ________________________

(FSW)

Size estimate FSW, lower bound of CI 1 ________________________

Size estimate FSW, upper bound of CI 1 ________________________

Specific region for size estimate 1 ________________________

Method for size estimate 1

- ◯ Service Multiplier
- ◯ Unique object multiplier
- ◯ Capture-recapture
- ◯ Network scale-up
- ◯ Wisdom of the crowds
- ◯ Delphi
- ◯ Program estimate
- ◯ General population survey
- ◯ Mapping and enumeration
- ◯ Reverse tracking method
- ◯ Literature Review
- ◯ Other

Size estimate 2 ______________________

(FSW)

Size estimate FSW, lower bound of CI 2 ______________________

Size estimate FSW, upper bound of CI 2 ______________________

Specific region for size estimate 2 ______________________

Method for size estimate 2
- ◯ Service Multiplier
- ◯ Unique object multiplier
- ◯ Capture-recapture
- ◯ Network scale-up
- ◯ Wisdom of the crowds
- ◯ Delphi
- ◯ Program estimate
- ◯ General population survey
- ◯ Mapping and enumeration
- ◯ Reverse tracking method
- ◯ Literature Review
- ◯ Other

Size estimate 3 ______________________

(FSW)

Size estimate FSW, lower bound of CI 3 ______________________

Size estimate FSW, upper bound of CI 3 ______________________

Specific region for size estimate 3 ______________________

Method for size estimate 3
- ◯ Service Multiplier
- ◯ Unique object multiplier
- ◯ Capture-recapture
- ◯ Network scale-up
- ◯ Wisdom of the crowds
- ◯ Delphi
- ◯ Program estimate
- ◯ General population survey
- ◯ Mapping and enumeration
- ◯ Reverse tracking method
- ◯ Literature Review
- ◯ Other

Size estimate 4 ______________________

(FSW)

Size estimate FSW, lower bound of CI 4 ______________________

Size estimate FSW, upper bound of CI 4 ______________________

Specific region for size estimate 4 ______________________

Method for size estimate 4

- ◯ Service Multiplier
- ◯ Unique object multiplier
- ◯ Capture-recapture
- ◯ Network scale-up
- ◯ Wisdom of the crowds
- ◯ Delphi
- ◯ Program estimate
- ◯ General population survey
- ◯ Mapping and enumeration
- ◯ Reverse tracking method
- ◯ Literature Review
- ◯ Other

Notes __________________________________

VIOLENCE

Violence denominator __________________________________

Number experienced physical violence __________________________________

(FSW)

Number experienced physical violence, lower bound of CI - calculated __________________________________

(FSW)

Number experienced physical violence, upper bound of CI - calculated __________________________________

(FSW)

Number experienced sexual violence __________________________________

(FSW)

Number experienced sexual violence, lower bound of CI - calculated __________________________________

(FSW)

Number experienced sexual violence, upper bound of CI - calculated __________________________________

(FSW)

Number experienced initimate partner violence __________________________________

(FSW)

Number experienced intimate partner violence, lower bound of CI - calculated ______________________

(FSW)

Number experienced intimate partner violence, upper bound of CI - calculated ______________________

(FSW)

FSW SPECIFIC INDICATORS

FSW specific variables denominator ______________________

Number who have consistent condom use ______________________

(FSW)

Number who have consistent condom use, lower bound of CI - calculated ______________________

(FSW)

Number who have consistent condom use, upper bound of CI - calculated ______________________

(FSW)

Consistent condom use - method ______________________

(FSW)

Number denied health services ______________________

(FSW)

Number denied health services, lower bound of CI - calculated ________________________

(FSW)

Number denied health services, upper bound of CI - calculated ________________________

(FSW)

Number afraid to seek health services ________________________

(FSW)

Number afraid to seek health services, lower bound of CI - calculated ________________________

(FSW)

Number afraid to seek health services, upper bound of CI - calculated ________________________

(FSW)

MEN WHO HAVE SEX WITH MEN

Sample size of MSM ______________________

BURDEN OF HIV (MSM)

Prevalence of HIV in sample (count) ______________________

(MSM)

Prevalence, lower bound of CI - reported ______________________

(MSM)

Prevalence, upper bound of CI - reported ______________________

(MSM)

Prevalence, lower bound of CI - calculated ______________________

(MSM)

Prevalence, upper bound of CI - calculated ______________________

(MSM)

Incidence of HIV in sample (count) ______________________

(MSM)

Incidence, lower bound of CI - reported ______________________

(MSM)

Incidence, upper bound of CI - reported ______________________

(MSM)

Incidence, lower bound of CI - calculated ______________________

(MSM)

Incidence, upper bound of CI - calculated ______________________

(MSM)

Notes, burden of disease

____

(MSM)

HIV PREVENTION CASCADE

Prevention cascade denominator (number of HIV negative) ____________________________

Number of HIV negative individuals tested for HIV ____________________________

(MSM)

Number of HIV negative individuals tested for HIV, lower bound of CI - calculated ____________________________

(MSM)

Number of HIV negative individuals tested for HIV, upper bound of CI - calculated ____________________________

(MSM)

Number tested negative ____________________________

(MSM)

Number tested negative, lower bound of CI - calculated ____________________________

(MSM)

Number tested negative, upper bound of CI - calculated ____________________________

(MSM)

Number with knowledge of HIV prevention ____________________________

(MSM)

Number with knowledge of HIV prevention, lower bound of CI - calculated ____________________________

(MSM)

Number with knowledge of HIV prevention, upper bound of CI - calculated

(MSM)

Number who report condom availability

(MSM)

Number who report condom availability, lower bound of CI - calculated

(MSM)

Number who report condom availability, upper bound of CI - calculated

(MSM)

Number who report PrEP availability

(MSM)

Number who report PrEP availability, lower bound of CI - calculated

(MSM)

Number who report PrEP availability, upper bound of CI - calculated

(MSM)

Notes, prevention cascade

(MSM)

HIV TREATMENT CASCADE

Treatment cascade denominator ________________________

Number tested (ever) ________________________

(MSM)

Number tested (ever), lower bound of CI - calculated ________________________

(MSM)

Number tested (ever), upper bound of CI - calculated ________________________

(MSM)

Number tested in last 12 months ________________________

(MSM)

Number tested in last 12 months, lower bound of CI - calculated ________________________

(MSM)

Number tested in last 12 months, upper bound of CI - calculated ________________________

(MSM)

Number previously diagnosed ________________________

(MSM)

Number previously diagnosed, lower bound of CI - calculated ________________________

(MSM)

Number previously diagnosed, upper bound of CI - calculated ________________________

(MSM)

Number linked to HIV care ________________________

(MSM)

Number linked to HIV care, lower bound of CI - calculated ________________________________

(MSM)

Number linked to HIV care, upper bound of CI - calculated ________________________________

(MSM)

Number retained in care ________________________________

(MSM)

Number retained in care, lower bound of CI - calculated ________________________________

(MSM)

Number retained in care, upper bound of CI - calculated ________________________________

(MSM)

Number on treatment ________________________________

(MSM)

Number on treatment, lower bound of CI - calculated ________________________________

(MSM)

Number on treatment, upper bound of CI - calculated ________________________________

(MSM)

Number adherent/undetectable ________________________________

(MSM)

Number adherent/undetectable, lower bound of CI - calculated ________________________________

(MSM)

Number adherent/undetectable, upper bound of CI - calculated ________________________

(MSM)

Notes, treatment cascade ______________________________

(MSM)

POPULATION SIZE ESTIMATES

Size estimate 1 ________________________

(MSM)

Size estimate MSM, lower bound of CI 1 ________________________

Size estimate MSM, upper bound of CI 1 ________________________

Specific region for size estimate 1 ________________________

Method for size estimate 1
- ◯ Service Multiplier
- ◯ Unique object multiplier
- ◯ Capture-recapture
- ◯ Network scale-up
- ◯ Wisdom of the crowds
- ◯ Delphi
- ◯ Program estimate
- ◯ General population survey
- ◯ Mapping and enumeration
- ◯ Reverse tracking method
- ◯ Literature Review
- ◯ Other

Size estimate 2 ________________________

(MSM)

Size estimate MSM, lower bound of CI 2 ________________________

Size estimate MSM, upper bound of CI 2 ________________________

Specific region for size estimate 2 ________________________

Method for size estimate 2

- ◯ Service Multiplier
- ◯ Unique object multiplier
- ◯ Capture-recapture
- ◯ Network scale-up
- ◯ Wisdom of the crowds
- ◯ Delphi
- ◯ Program estimate
- ◯ General population survey
- ◯ Mapping and enumeration
- ◯ Reverse tracking method
- ◯ Literature Review
- ◯ Other

Size estimate 3 ______________________________

(MSM)

Size estimate MSM, lower bound of CI 3 ______________________________

Size estimate MSM, upper bound of CI 3 ______________________________

Specific region for size estimate 3 ______________________________

Method for size estimate 3

- ◯ Service Multiplier
- ◯ Unique object multiplier
- ◯ Capture-recapture
- ◯ Network scale-up
- ◯ Wisdom of the crowds
- ◯ Delphi
- ◯ Program estimate
- ◯ General population survey
- ◯ Mapping and enumeration
- ◯ Reverse tracking method
- ◯ Literature Review
- ◯ Other

Size estimate 4 ______________________________

(MSM)

Size estimate MSM, lower bound of CI 4 ______________________________

Size estimate MSM, upper bound of CI 4 ______________________________

Specific region for size estimate 4 ______________________________

Method for size estimate 4

- ◯ Service Multiplier
- ◯ Unique object multiplier
- ◯ Capture-recapture
- ◯ Network scale-up
- ◯ Wisdom of the crowds
- ◯ Delphi
- ◯ Program estimate
- ◯ General population survey
- ◯ Mapping and enumeration
- ◯ Reverse tracking method
- ◯ Literature Review
- ◯ Other

Notes ______________________________

VIOLENCE

Violence denominator ______________________

Number experienced physical violence ______________________

(MSM)

Number experienced physical violence, lower bound of CI - calculated ______________________

(MSM)

Number experienced physical violence, upper bound of CI - calculated ______________________

(MSM)

Number experienced sexual violence ______________________

(MSM)

Number experienced sexual violence, lower bound of CI - calculated ______________________

(MSM)

Number experienced sexual violence, upper bound of CI - calculated ______________________

(MSM)

Number experienced initimate partner violence ______________________

(MSM)

Number experienced intimate partner violence, lower bound of CI - calculated ______________________

(MSM)

Number experienced intimate partner violence, upper bound of CI - calculated ______________________

(MSM)

MSM SPECIFIC INDICATORS

MSM specific variables denominator ______________________

Number who have consistent condom use ______________________

(MSM)

Number who have consistent condom use, lower bound of CI - calculated ______________________

(MSM)

Number who have consistent condom use, upper bound of CI - calculated ______________________

(MSM)

Consistent condom use - method ______________________

(MSM)

Number denied health services ______________________

(MSM)

Number denied health services, lower bound of CI - calculated ______________________

(MSM)

Number denied health services, upper bound of CI - calculated ______________________

(MSM)

Number afraid to seek health services ________________________________

(MSM)

Number afraid to seek health services, lower bound of CI - calculated ________________________________

(MSM)

Number afraid to seek health services, upper bound of CI - calculated ________________________________

(MSM)

PEOPLE WHO USE DRUGS

Sample size of PWUD ________________________________

BURDEN OF HIV (PWUD)

Prevalence of HIV in sample (count) ________________________________

(PWUD)

Prevalence, lower bound of CI - reported ________________________________

(PWUD)

Prevalence, upper bound of CI - reported ________________________________

(PWUD)

Prevalence, lower bound of CI - calculated ________________________________

(PWUD)

Prevalence, upper bound of CI - calculated ________________________

(PWUD)

Incidence of HIV in sample (count) ________________________

(PWUD)

Incidence, lower bound of CI - reported ________________________

(PWUD)

Incidence, upper bound of CI - reported ________________________

(PWUD)

Incidence, lower bound of CI - calculated ________________________

(PWUD)

Incidence, upper bound of CI - calculated ________________________

(PWUD)

Notes, burden of disease

____

(PWUD)

HIV PREVENTION CASCADE

Prevention cascade denominator (number of HIV negative) ________________________

Number of HIV negative individuals tested for HIV ________________________

(PWUD)

Number of HIV negative individuals tested for HIV, lower bound of CI - calculated ________________________

(PWUD)

Number of HIV negative individuals tested for HIV, upper bound of CI - calculated ________________________

(PWUD)

Number tested negative ______________________

(PWUD)

Number tested negative, lower bound of CI - calculated ______________________

(PWUD)

Number tested negative, upper bound of CI - calculated ______________________

(PWUD)

Number with knowledge of HIV prevention ______________________

(PWUD)

Number with knowledge of HIV prevention, lower bound of CI - calculated ______________________

(PWUD)

Number with knowledge of HIV prevention, upper bound of CI - calculated ______________________

(PWUD)

Number who report condom availability ______________________

(PWUD)

Number who report condom availability, lower bound of CI - calculated ______________________

(PWUD)

Number who report condom availability, upper bound of CI - calculated ______________________

(PWUD)

Number who report PrEP availability ______________________

(PWUD)

Number who report PrEP availability, lower bound of CI - calculated ______________________

(PWUD)

Number who report PrEP availability, upper bound of CI - calculated ______________________

(PWUD)

Notes, prevention cascade ______________________

(PWUD)

HIV TREATMENT CASCADE

Treatment cascade denominator ______________________

Number tested (ever) ______________________

(PWUD)

Number tested (ever), lower bound of CI - calculated ______________________

(PWUD)

Number tested (ever), upper bound of CI - calculated ______________________

(PWUD)

Number tested in last 12 months ______________________

(PWUD)

Number tested in last 12 months, lower bound of CI - calculated ______________________

(PWUD)

Number tested in last 12 months, upper bound of CI - calculated ______________________

(PWUD)

Number previously diagnosed ______________________

(PWUD)

Number previously diagnosed, lower bound of CI - calculated ______________________

(PWUD)

Number previously diagnosed, upper bound of CI - calculated ______________________

(PWUD)

Number linked to HIV care ______________________

(PWUD)

Number linked to HIV care, lower bound of CI - calculated ______________________

(PWUD)

Number linked to HIV care, upper bound of CI - calculated ______________________

(PWUD)

Number retained in care ______________________

(PWUD)

Number retained in care, lower bound of CI - calculated ______________________

(PWUD)

Number retained in care, upper bound of CI - calculated ______________________

(PWUD)

Number on treatment ______________________

(PWUD)

Number on treatment, lower bound of CI - calculated ______________________

(PWUD)

Number on treatment, upper bound of CI - calculated ________________________

(PWUD)

Number adherent/undetectable ________________________

(PWUD)

Number adherent/undetectable, lower bound of CI - calculated ________________________

(PWUD)

Number adherent/undetectable, upper bound of CI - calculated ________________________

(PWUD)

Notes, treatment cascade

____

(PWUD)

POPULATION SIZE ESTIMATES

Size estimate 1 ________________________

(PWUD)

Size estimate PWUD, lower bound of CI 1 ________________________

Size estimate PWUD, upper bound of CI 1 ________________________

Specific region for size estimate 1 ________________________

Method for size estimate 1

- ◯ Service Multiplier
- ◯ Unique object multiplier
- ◯ Capture-recapture
- ◯ Network scale-up
- ◯ Wisdom of the crowds
- ◯ Delphi
- ◯ Program estimate
- ◯ General population survey
- ◯ Mapping and enumeration
- ◯ Reverse tracking method
- ◯ Literature Review
- ◯ Other

Size estimate 2 ______________________________

(PWUD)

Size estimate PWUD, lower bound of CI 2 ______________________________

Size estimate PWUD, upper bound of CI 2 ______________________________

Specific region for size estimate 2 ______________________________

Method for size estimate 2
- ◯ Service Multiplier
- ◯ Unique object multiplier
- ◯ Capture-recapture
- ◯ Network scale-up
- ◯ Wisdom of the crowds
- ◯ Delphi
- ◯ Program estimate
- ◯ General population survey
- ◯ Mapping and enumeration
- ◯ Reverse tracking method
- ◯ Literature Review
- ◯ Other

Size estimate 3 ______________________________

(PWUD)

Size estimate PWUD, lower bound of CI 3 ______________________________

Size estimate PWUD, upper bound of CI 3 ______________________________

Specific region for size estimate 3 ______________________________

Method for size estimate 3
- ◯ Service Multiplier
- ◯ Unique object multiplier
- ◯ Capture-recapture
- ◯ Network scale-up
- ◯ Wisdom of the crowds
- ◯ Delphi
- ◯ Program estimate
- ◯ General population survey
- ◯ Mapping and enumeration
- ◯ Reverse tracking method
- ◯ Literature Review
- ◯ Other

Size estimate 4 ______________________________

(PWUD)

Size estimate PWUD, lower bound of CI 4 ______________________________

Size estimate PWUD, upper bound of CI 4 ______________________________

Specific region for size estimate 4 ______________________________

Method for size estimate 4

◯ Service Multiplier
◯ Unique object multiplier
◯ Capture-recapture
◯ Network scale-up
◯ Wisdom of the crowds
◯ Delphi
◯ Program estimate
◯ General population survey
◯ Mapping and enumeration
◯ Reverse tracking method
◯ Literature Review
◯ Other

Notes ________________________________

VIOLENCE

Violence denominator ________________________________

Number experienced physical violence ________________________________

(PWUD)

Number experienced physical violence, lower bound of CI - calculated ________________________________

(PWUD)

Number experienced physical violence, upper bound of CI - calculated ________________________________

(PWUD)

Number experienced sexual violence ________________________________

(PWUD)

Number experienced sexual violence, lower bound of CI - calculated ________________________________

(PWUD)

Number experienced sexual violence, upper bound of CI - calculated ________________________________

(PWUD)

Number experienced initimate partner violence ________________________________

(PWUD)

Number experienced intimate partner violence, lower bound of CI - calculated

(PWUD)

Number experienced intimate partner violence, upper bound of CI - calculated

(PWUD)

PWUD SPECIFIC INDICATORS

PWUD specific variables denominator

Number who have consistent condom use

(PWUD)

Number who have consistent condom use, lower bound of CI - calculated

(PWUD)

Number who have consistent condom use, upper bound of CI - calculated

(PWUD)

Consistent condom use - method

(PWUD)

Number denied health services

(PWUD)

Number denied health services, lower bound of CI - calculated
____

(PWUD)

Number denied health services, upper bound of CI - calculated
____

(PWUD)

Number afraid to seek health services
____

(PWUD)

Number afraid to seek health services, lower bound of CI - calculated
____

(PWUD)

Number afraid to seek health services, upper bound of CI - calculated
____

(PWUD)

Number of needles per injector
____

(PWUD)

Number of needles per injector, lower bound of CI - calculated ________________________________

(PWUD)

Number of needles per injector, upper bound of CI - calculated ________________________________

(PWUD)

TRANSGENDER PEOPLE

Sample size of Transgender ________________________________

BURDEN OF HIV (Trans)

Prevalence of HIV in sample (count) ________________________________

(Trans)

Prevalence, lower bound of CI - reported ________________________________

(Trans)

Prevalence, upper bound of CI - reported ________________________________

(Trans)

Prevalence, lower bound of CI - calculated ________________________________

(Trans)

Prevalence, upper bound of CI - calculated ________________________________

(Trans)

Incidence of HIV in sample (count) ________________________________

(Trans)

Incidence, lower bound of CI - reported ________________

(Trans)

Incidence, upper bound of CI - reported ________________

(Trans)

Incidence, lower bound of CI - calculated ________________

(Trans)

Incidence, upper bound of CI - calculated ________________

(Trans)

Notes, burden of disease

____

(Trans)

HIV PREVENTION CASCADE

Prevention cascade denominator (number of HIV negative) ________________

Number of HIV negative individuals tested for HIV ________________

(Trans)

Number of HIV negative individuals tested for HIV, lower bound of CI - calculated ________________

(Trans)

Number of HIV negative individuals tested for HIV, upper bound of CI - calculated ________________

(Trans)

Number tested negative ________________

(Trans)

Number tested negative, lower bound of CI - calculated ________________

(Trans)

Number tested negative, upper bound of CI - calculated ________________________________

(Trans)

Number with knowledge of HIV prevention ________________________________

(Trans)

Number with knowledge of HIV prevention, lower bound of CI - calculated ________________________________

(Trans)

Number with knowledge of HIV prevention, upper bound of CI - calculated ________________________________

(Trans)

Number who report condom availability ________________________________

(Trans)

Number who report condom availability, lower bound of CI - calculated ________________________________

(Trans)

Number who report condom availability, upper bound of CI - calculated ________________________________

(Trans)

Number who report PrEP availability ________________________________

(Trans)

Number who report PrEP availability, lower bound of CI - calculated ________________________________

(Trans)

Number who report PrEP availability, upper bound of CI - calculated ________________________________

(Trans)

Notes, prevention cascade

(Trans)

HIV TREATMENT CASCADE

Treatment cascade denominator

Number tested (ever)

(Trans)

Number tested (ever), lower bound of CI - calculated

(Trans)

Number tested (ever), upper bound of CI - calculated

(Trans)

Number tested in last 12 months

(Trans)

Number tested in last 12 months, lower bound of CI - calculated

(Trans)

Number tested in last 12 months, upper bound of CI - calculated

(Trans)

Number previously diagnosed

(Trans)

Number previously diagnosed, lower bound of CI - calculated

(Trans)

Number previously diagnosed, upper bound of CI - calculated ______________________

(Trans)

Number linked to HIV care ______________________

(Trans)

Number linked to HIV care, lower bound of CI - calculated ______________________

(Trans)

Number linked to HIV care, upper bound of CI - calculated ______________________

(Trans)

Number retained in care ______________________

(Trans)

Number retained in care, lower bound of CI - calculated ______________________

(Trans)

Number retained in care, upper bound of CI - calculated ______________________

(Trans)

Number on treatment ______________________

(Trans)

Number on treatment, lower bound of CI - calculated ______________________

(Trans)

Number on treatment, upper bound of CI - calculated ______________________

(Trans)

Number adherent/undetectable ______________________

(Trans)

Number adherent/undetectable, lower bound of CI - calculated ______________________

(Trans)

Number adherent/undetectable, upper bound of CI - calculated ______________________

(Trans)

Notes, treatment cascade

____

(Trans)

POPULATION SIZE ESTIMATES

Size estimate 1 ______________________

(Trans)

Size estimate Trans, lower bound of CI 1 ______________________

Size estimate Trans, upper bound of CI 1 ______________________

Specific region for size estimate 1 ______________________

Method for size estimate 1
- ◯ Service Multiplier
- ◯ Unique object multiplier
- ◯ Capture-recapture
- ◯ Network scale-up
- ◯ Wisdom of the crowds
- ◯ Delphi
- ◯ Program estimate
- ◯ General population survey
- ◯ Mapping and enumeration
- ◯ Reverse tracking method
- ◯ Literature Review
- ◯ Other

Size estimate 2 ______________________

(Trans)

Size estimate Trans, lower bound of CI 2 ______________________

Size estimate Trans, upper bound of CI 2 ______________________

Specific region for size estimate 2 ______________________

Method for size estimate 2

- ◯ Service Multiplier
- ◯ Unique object multiplier
- ◯ Capture-recapture
- ◯ Network scale-up
- ◯ Wisdom of the crowds
- ◯ Delphi
- ◯ Program estimate
- ◯ General population survey
- ◯ Mapping and enumeration
- ◯ Reverse tracking method
- ◯ Literature Review
- ◯ Other

Size estimate 3 ______________________

(Trans)

Size estimate Trans, lower bound of CI 3 ______________________

Size estimate Trans, upper bound of CI 3 ______________________

Specific region for size estimate 3 ______________________

Method for size estimate 3

- ◯ Service Multiplier
- ◯ Unique object multiplier
- ◯ Capture-recapture
- ◯ Network scale-up
- ◯ Wisdom of the crowds
- ◯ Delphi
- ◯ Program estimate
- ◯ General population survey
- ◯ Mapping and enumeration
- ◯ Reverse tracking method
- ◯ Literature Review
- ◯ Other

Size estimate 4 ______________________

(Trans)

Size estimate Trans, lower bound of CI 4 ______________________

Size estimate Trans, upper bound of CI 4 ______________________

Specific region for size estimate 4 ______________________

Method for size estimate 4

- ◯ Service Multiplier
- ◯ Unique object multiplier
- ◯ Capture-recapture
- ◯ Network scale-up
- ◯ Wisdom of the crowds
- ◯ Delphi
- ◯ Program estimate
- ◯ General population survey
- ◯ Mapping and enumeration
- ◯ Reverse tracking method
- ◯ Literature Review
- ◯ Other

Notes ______________________

VIOLENCE

Violence denominator __________________________________

Number experienced physical violence __________________________________

(Trans)

Number experienced physical violence, lower bound of CI - calculated __________________________________

(Trans)

Number experienced physical violence, upper bound of CI - calculated __________________________________

(Trans)

Number experienced sexual violence __________________________________

(Trans)

Number experienced sexual violence, lower bound of CI - calculated __________________________________

(Trans)

Number experienced sexual violence, upper bound of CI - calculated __________________________________

(Trans)

Number experienced initimate partner violence __________________________________

(Trans)

Number experienced intimate partner violence, lower bound of CI - calculated __________________________________

(Trans)

Number experienced intimate partner violence, upper bound of CI - calculated __________________________________

(Trans)

Trans SPECIFIC INDICATORS

Trans specific variables denominator ________________________________

Number who have consistent condom use ________________________________

(Trans)

Number who have consistent condom use, lower bound of CI - calculated ________________________________

(Trans)

Number who have consistent condom use, upper bound of CI - calculated ________________________________

(Trans)

Consistent condom use - method ________________________________

(Trans)

Number denied health services ________________________________

(Trans)

Number denied health services, lower bound of CI - calculated ________________________________

(Trans)

Number denied health services, upper bound of CI - calculated ________________________________

(Trans)

Number afraid to seek health services ________________

(Trans)

Number afraid to seek health services, lower bound of CI - calculated ________________

(Trans)

Number afraid to seek health services, upper bound of CI - calculated ________________

(Trans)

INCARCERATE PEOPLE

Sample size of incarcerated ________________

BURDEN OF HIV (incarcerated)

Prevalence of HIV in sample (count) ________________

(incarcerated)

Prevalence, lower bound of CI - reported ________________

(incarcerated)

Prevalence, upper bound of CI - reported ________________

(incarcerated)

Prevalence, lower bound of CI - calculated ________________

(incarcerated)

Prevalence, upper bound of CI - calculated ______________________

(incarcerated)

Incidence of HIV in sample (count) ______________________

(incarcerated)

Incidence, lower bound of CI - reported ______________________

(incarcerated)

Incidence, upper bound of CI - reported ______________________

(incarcerated)

Incidence, lower bound of CI - calculated ______________________

(incarcerated)

Incidence, upper bound of CI - calculated ______________________

(incarcerated)

Notes, burden of disease

____

(incarcerated)

HIV PREVENTION CASCADE

Prevention cascade denominator (number of HIV negative) ______________________

Number of HIV negative individuals tested for HIV ______________________

(incarcerated)

Number of HIV negative individuals tested for HIV, lower bound of CI - calculated ______________________

(incarcerated)

Number of HIV negative individuals tested for HIV, upper bound of CI - calculated ______________________

(incarcerated)

Number tested negative ______________________

(incarcerated)

Number tested negative, lower bound of CI - calculated ______________________

(incarcerated)

Number tested negative, upper bound of CI - calculated ______________________

(incarcerated)

Number with knowledge of HIV prevention ______________________

(incarcerated)

Number with knowledge of HIV prevention, lower bound of CI - calculated ______________________

(incarcerated)

Number with knowledge of HIV prevention, upper bound of CI - calculated ______________________

(incarcerated)

Number who report condom availability ______________________

(incarcerated)

Number who report condom availability, lower bound of CI - calculated ______________________

(incarcerated)

Number who report condom availability, upper bound of CI - calculated ______________________

(incarcerated)

Number who report PrEP availability ______________________

(incarcerated)

Number who report PrEP availability, lower bound of CI - calculated ______________________

(incarcerated)

Number who report PrEP availability, upper bound of CI - calculated ______________________

(incarcerated)

Notes, prevention cascade

____

(incarcerated)

HIV TREATMENT CASCADE

Treatment cascade denominator ______________________

Number tested (ever) ______________________

(incarcerated)

Number tested (ever), lower bound of CI - calculated ______________________

(incarcerated)

Number tested (ever), upper bound of CI - calculated ______________________

(incarcerated)

Number tested in last 12 months ______________________

(incarcerated)

Number tested in last 12 months, lower bound of CI - calculated ______________________

(incarcerated)

Number tested in last 12 months, upper bound of CI - calculated ______________________

(incarcerated)

Number previously diagnosed ______________________

(incarcerated)

Number previously diagnosed, lower bound of CI - calculated ______________________

(incarcerated)

Number previously diagnosed, upper bound of CI - calculated ______________________

(incarcerated)

Number linked to HIV care ______________________

(incarcerated)

Number linked to HIV care, lower bound of CI - calculated ______________________

(incarcerated)

Number linked to HIV care, upper bound of CI - calculated ______________________

(incarcerated)

Number retained in care ______________________

(incarcerated)

Number retained in care, lower bound of CI - calculated ______________________

(incarcerated)

Number retained in care, upper bound of CI - calculated ______________________

(incarcerated)

Number on treatment ______________________

(incarcerated)

Number on treatment, lower bound of CI - calculated ______________________

(incarcerated)

Number on treatment, upper bound of CI - calculated ________________________________

(incarcerated)

Number adherent/undetectable ________________________________

(incarcerated)

Number adherent/undetectable, lower bound of CI - calculated ________________________________

(incarcerated)

Number adherent/undetectable, upper bound of CI - calculated ________________________________

(incarcerated)

Notes, treatment cascade

____

(incarcerated)

POPULATION SIZE ESTIMATES

Size estimate 1 ________________________________

(incarcerated)

Size estimate incarcerated, lower bound of CI 1 ________________________________

Size estimate incarcerated, upper bound of CI 1 ________________________________

Specific region for size estimate 1 ________________________________

Method for size estimate 1

- ◯ Service Multiplier
- ◯ Unique object multiplier
- ◯ Capture-recapture
- ◯ Network scale-up
- ◯ Wisdom of the crowds
- ◯ Delphi
- ◯ Program estimate
- ◯ General population survey
- ◯ Mapping and enumeration
- ◯ Reverse tracking method
- ◯ Literature Review
- ◯ Other

Size estimate 2 ______________________

(incarcerated)

Size estimate incarcerated, lower bound of CI 2 ______________________

Size estimate incarcerated, upper bound of CI 2 ______________________

Specific region for size estimate 2 ______________________

Method for size estimate 2
- ◯ Service Multiplier
- ◯ Unique object multiplier
- ◯ Capture-recapture
- ◯ Network scale-up
- ◯ Wisdom of the crowds
- ◯ Delphi
- ◯ Program estimate
- ◯ General population survey
- ◯ Mapping and enumeration
- ◯ Reverse tracking method
- ◯ Literature Review
- ◯ Other

Size estimate 3 ______________________

(incarcerated)

Size estimate incarcerated, lower bound of CI 3 ______________________

Size estimate incarcerated, upper bound of CI 3 ______________________

Specific region for size estimate 3 ______________________

Method for size estimate 3
- ◯ Service Multiplier
- ◯ Unique object multiplier
- ◯ Capture-recapture
- ◯ Network scale-up
- ◯ Wisdom of the crowds
- ◯ Delphi
- ◯ Program estimate
- ◯ General population survey
- ◯ Mapping and enumeration
- ◯ Reverse tracking method
- ◯ Literature Review
- ◯ Other

Size estimate 4 ______________________

(incarcerated)

Size estimate incarcerated, lower bound of CI 4 ______________________

Size estimate incarcerated, upper bound of CI 4 ______________________

Specific region for size estimate 4 ______________________

Method for size estimate 4

- ◯ Service Multiplier
- ◯ Unique object multiplier
- ◯ Capture-recapture
- ◯ Network scale-up
- ◯ Wisdom of the crowds
- ◯ Delphi
- ◯ Program estimate
- ◯ General population survey
- ◯ Mapping and enumeration
- ◯ Reverse tracking method
- ◯ Literature Review
- ◯ Other

Notes __________________________________

VIOLENCE

Violence denominator __________________________________

Number experienced physical violence __________________________________

(incarcerated)

Number experienced physical violence, lower bound of CI - calculated __________________________________

(incarcerated)

Number experienced physical violence, upper bound of CI - calculated __________________________________

(incarcerated)

Number experienced sexual violence __________________________________

(incarcerated)

Number experienced sexual violence, lower bound of CI - calculated __________________________________

(incarcerated)

Number experienced sexual violence, upper bound of CI - calculated __________________________________

(incarcerated)

Number experienced initimate partner violence __________________________________

(incarcerated)

Number experienced intimate partner violence, lower bound of CI - calculated ______________________________

(incarcerated)

Number experienced intimate partner violence, upper bound of CI - calculated ______________________________

(incarcerated)

---

incarcerated SPECIFIC INDICATORS

incarcerated specific variables denominator ______________________________

Number who have consistent condom use ______________________________

(incarcerated)

Number who have consistent condom use, lower bound of CI - calculated ______________________________

(incarcerated)

Number who have consistent condom use, upper bound of CI - calculated ______________________________

(incarcerated)

Consistent condom use - method ______________________________

(incarcerated)

Number denied health services ______________________________

(incarcerated)

Number denied health services, lower bound of CI - calculated ______________________

(incarcerated)

Number denied health services, upper bound of CI - calculated ______________________

(incarcerated)

Number afraid to seek health services ______________________

(incarcerated)

Number afraid to seek health services, lower bound of CI - calculated ______________________

(incarcerated)

Number afraid to seek health services, upper bound of CI - calculated ______________________

(incarcerated)
