# Supplementary material for: HIV-related data among key populations to inform evidence-based responses: protocol of a systematic review
Source: Syst Rev. 2018 Dec 3;7:220. doi: 10.1186/s13643-018-0894-3 (PMC6278072; doi:10.1186/s13643-018-0894-3)
Supplement: Supplementary file 3 — Provides the quality assessment tool used to evaluate data points for prevalence and incidence of HIV, the treatment cascade, and population size estimates. (DOCX 32 kb) [file 13643_2018_894_MOESM3_ESM.docx]

**Quality Assessment Tool: Detailed Instructions**

*Adapted from NHLBI quality assessment tool*

There are three scores: good, fair, and poor. For each individual section of the main tool, design and implementation, the study will receive a score. The study will also receive an individual score for each of the indicator-specific questions that are relevant (prevalence, incidence, HIV care continuum, and population size estimates). *

*When reviewing a study with multiple published papers associated with the same sample, the evidence should be reviewed together. A primary paper should be selected and the other published papers will be used to fill in gaps. Some indications that a paper might serve as the “primary paper” would include if it presents data that was the primary aim of the original study, if the other papers cite it for methodological details, if it was published first, etc.

The study gets 1 point from each “yes” it receives. The scoring is as follows for each section:

| **Section** | **TOTAL PTS** | **GOOD** | **FAIR** | **POOR** |
| --- | --- | --- | --- | --- |
| **OVERALL** | | | | |
| Study design | 4 | 4 | 2-3 | 0-1 |
| Implementation | 3 | 3 | 2 | 0-1 |
| **INDICATOR-SPECIFIC** |  |  |  |  |
| Prevalence of HIV | 6 | 6 | 3-5 | 0-2 |
| Incidence of HIV | 9 | 8-9 | 5-7 | 0-4 |
| HIV Care Continuum | 9 | 8-9 | 5-7 | 0-4 |
| Population Size Estimates | 7 | 7 | 4-6 | 0-3 |

The scoring that incorporates overall study score (Study design and implementation) works as follows:

| **Section** | **TOTAL PTS** | **GOOD** | **FAIR** | **POOR** |
| --- | --- | --- | --- | --- |
| Prevalence of HIV | 13 | 13 | 7-12 | 0-6 |
| Incidence of HIV | 16 | 15-16 | 7-14 | 0-6 |
| HIV Care Continuum | 16 | 15-16 | 7-14 | 0-6 |
| Population Size Estimates | 14 | 14 | 8-13 | 0-7 |

**Quality Assessment Tool**

ARTICLE UNIQUE ID (Primary): _ _ _ _ _ _ _ _ _ _ _

ARTICLE TITLE: ______________________________________________

PUBLICATION YEAR: _ _ _ _

COHORT ID: _ _ _ _ _ _ _ _ _ _ _ _ _

OTHER PAPERS FROM THE SAME STUDY:

_ _ _ _ _ _ _ _ _ _ _ _ _ _ _ _ _ _ _ _ _ _ _ _ _ _

_ _ _ _ _ _ _ _ _ _ _ _ _ _ _ _ _ _ _ _ _ _ _ _ _ _

_ _ _ _ _ _ _ _ _ _ _ _ _ _ _ _ _ _ _ _ _ _ _ _ _ _

_ _ _ _ _ _ _ _ _ _ _ _ _ _ _ _ _ _ _ _ _ _ _ _ _ _

**Quantitative, empirical data?** Study design score: ______

+

Study implementation score: ______

=**Overall study score**: ______

AND

**Prevalence of HIV data** Prevalence score: ______

OR

**Incidence of HIV data** Incidence score: ______

OR

**HIV care continuum data** Care Continuum score: ______

- HIV testing
- Diagnosis
- Treatment
- Viral suppression

OR

**Population size estimation data** PSE score: ______

**Include in quality assessment?** YES NO

**Study Design (total pts: 4)**

| Criteria | **Yes** | **No** | **Add. paper utilized (Specify):** |
| --- | --- | --- | --- |
| 1. Was the research question or objective in this study clearly stated? |  |  |  |
| BRIEFLY STATE PRIMARY RESEARCH QUESTION: | | | |
| 1. Was the study population clearly defined? |  |  |  |
| 1. Will the study population defined answer the research question proposed (adequately represent the target population)? |  |  |  |
| 1. Was the sample size justified either through a power description, or variance and effect estimates? |  |  |  |

*1. Research question*: Did the authors describe their goal in conducting this research? Is it easy to understand what they were looking to find?

2 and 3. *Study population*: Did the authors describe the group of people from which the study participants were selected or recruited, using demographics, location, and time period? If you were to conduct this study again, would you know who to recruit, from where, and from what time period?

An example would be female sex workers 18 years or older who were recruited through venue-based sampling between December 17, 2015 and April 20, 2017. (**Person, place, time**)

Were the inclusion and exclusion criteria developed prior to recruitment or selection of the study population? Were the same underlying criteria used for all the subjects involved?

For question 3, will the study population proposed by the authors actually answer the question of interest? For example, if the authors are looking to understand viral load suppression among female sex workers, but the study design indicates only recruiting from clinics (where individuals are likely already linked to care), this study population may not adequately represent the target population.

4. *Sample size justification*: Did the authors present their reasons for selecting or recruiting the number of people included or analyzed? Do they note or discuss the statistical power or precision of estimates of the study? This question is about whether or not the study had enough participants to meaningful measure the outcome, or in the case of intervention studies to detect a difference if one exists.

A paragraph in the methods section may explain the sample size needed to estimate incidence or prevalence with a margin of error (e.g. +/- 2%) or to detect a hypothesized difference in outcomes. You may also find a discussion of power in the discussion section (such as the study had 85 percent power to detect a 20 percent increase in the rate of an outcome of interest, with a 2-sided alpha of 0.05). **Sometimes estimates of variance and/or estimates of effect size are given, instead of sample size calculations**. In any of these cases, the answer would be “yes.”

**Study Implementation (total pts: 3)**

| Criteria | **Yes** | **No** | **Add. paper utilized (Specify):** |
| --- | --- | --- | --- |
| 1. Was the proportion of people who agreed to participate reported? |  |  |  |
| 1. Was the proportion who agreed to participate at least 85%? |  |  |  |
| 1. Is there reason to believe that the participants enrolled are a representative sample of the source population (the population from which participants were recruited)? |  |  |  |

*1 and 2. Participation rate*

If fewer than 85% of eligible persons participated in the study, then there is concern that the study population does not adequately represent the target population. This increases the risk of bias. If the paper or a cited previous paper does not include mention of rate of refusals, the answer would be “NR.” For respondent-driven sampling, did the authors conduct any assessments of equilibrium or bottleneck effects?

3. *Representative sampling*

Were participants selected with equal probably of sampling from the source population? For example, if a representative set of venues is chosen from which to recruit participants (the source population), are participants recruited equally from these venues? One issue, for example, could arise if participants who are known to be living with HIV systematically decline to participate when approached.

**Quality Assessment Tool: Indicator-Specific Questions**

**Prevalence of HIV (total pts: 6)**

| Criteria | **Yes** | **No** | **Add. paper utilized (Specify):** |
| --- | --- | --- | --- |
| 1. Are the numerator (number living with HIV) and denominator reported? |  |  |  |
| 1. Was the **HIV outcome** measured using a biological test? |  |  |  |
| 1. Was the **HIV outcome** clearly defined? |  |  |  |
| 1. Was the **HIV outcome** assessed consistently across all study participants? |  |  |  |
| 1. Were the statistical methods used to assess **prevalence** appropriate? The statistical techniques must be appropriate to the data.* |  |  |  |
| 1. Have appropriate confidence intervals been reported? |  |  |  |

1. Are both the number of people living with HIV and total number of people enrolled in the study clearly reported? If only one reported, the answer would be “no.”

2. The rationale for this question is that in assessing prevalence, biological testing (“yes”) would lead to superior quality of data over self-reported (“no”) HIV prevalence. For HIV outcomes assessed by self-testing, outcome verification must be clear and the result must be verified.

3. Some criteria for determining if the HIV infection outcome was “clearly defined” include: how was HIV outcome measured (self-report, self-test, oral rapid test conducted by study, rapid blood test conducted by study, etc.) and what criteria or algorithm were used to determine a positive test result. In order for this question to receive an answer of “yes,” HIV outcome must be clearly defined.

4. In order for this question to receive an answer of “yes,” HIV outcome must have been assessed consistently across all study participants. For example, if data on HIV status is missing for a number of participants and this missingness was not accounted for, the answer would be “no.”

5.Were sampling weights included if appropriate? For time-location sampling and other venue-based sampling, were sampling weights for venues and individuals included if appropriate? For respondent-driven sampling (RDS) studies, were RDS adjustments included?

6. Some measure of uncertainty should be provided. For a “yes” response, appropriate confidence or credible intervals that take into account sampling design have been calculated around the estimate.

**Incidence of HIV (total pts: 9)**

| Criteria | **Yes** | **No** | **Add. paper utilized (Specify):** |
| --- | --- | --- | --- |
| 1. Are the numerator (number newly infected with HIV) and total amount of person-time at risk reported? |  |  |  |
| 1. Was the **HIV outcome** measured using a biological test? |  |  |  |
| 1. Was the **HIV outcome** clearly defined? |  |  |  |
| 1. Was the **HIV outcome** assessed consistently across all study participants? |  |  |  |
| 1. Were the statistical methods used to assess **HIV outcome** appropriate? The statistical techniques must be appropriate to the data.* |  |  |  |
| 1. Have actual confidence intervals been reported? |  |  |  |
| 1. Was the time frame sufficient so that one could reasonably expect to incident infections representative of the underlying incidence? |  |  |  |
| 1. By the end of the study, was retention reasonable? |  |  |  |
| 1. If dropout was greater than or equal to 10%, did the authors assess reasons for dropout or compare those who dropped out to those who remained in follow-up? |  |  |  |

1. Are both the number newly infected with HIV and the total amount of person-time at risk (or numbers of persons at risk over a time period) for infection clearly reported? If only one reported, the answer would be “no.”

2. The rationale for this question is that in assessing prevalence, biological testing (“yes”) would lead to superior quality of data over self-reported (“no”) HIV prevalence. For HIV outcomes assessed by self-testing, outcome verification must be clear and the result must be verified.

3. Some criteria for determining if the HIV infection outcome was “clearly defined” include: how was HIV outcome measured (self-report, self-test, oral rapid test conducted by study, rapid blood test conducted by study, etc.) and what criteria or algorithm were used to determine a positive test result. In order for this question to receive an answer of “yes,” HIV outcome must be clearly defined.

4. In order for this question to receive an answer of “yes,” HIV outcome must have been assessed consistently across all study participants. For example, if data on HIV status is missing for a number of participants and this missingness was not accounted for, the answer would be “no.”

5. Were sampling weights included if appropriate. For time-location sampling and other venue-based sampling, were biases by venue considered? For respondent-driven sampling studies, were RDS adjustments included? Was missingness adequately accounted for?

6. Some measure of uncertainty should be provided. For a “yes” response, appropriate confidence or credible intervals that take into account sampling design have been calculated around the estimate.

7. *Sufficient timeframe*: Is the study powered to assess incidence in the population of interest? For example, if the study is following along individuals to assess new HIV infections, such an observation may take several years or a sufficient amount of person-time.

Are the confidence intervals for incidence are narrow enough to make the incidence estimate meaningful? The follow up does not necessarily have to be long, but if you have a high incidence and lots of people, you could still have a reasonable estimate.

*8. Follow-up rate*: Is there any reason to believe that those who dropout are different from those who remained in follow-up?

9. If drop-out is greater than or equal to 10%, did the authors compare characteristics of those who dropped out with those who remained in follow-up? Did the authors look at what was associated with dropout?

**HIV Care Continuum (total pts: 9)**

| Criteria | **Yes** | **No** | **Add. paper utilized (Specify):** |
| --- | --- | --- | --- |
| 1. Are the numerators and denominators reported for each indicator *that was reported* of the care continuum? |  |  |  |
| 1. Were the **indicators of the care continuum** clearly defined? |  |  |  |
| 1. Were the **indicators of the care continuum** assessed consistently across all study participants? |  |  |  |
| 1. Were the statistical methods used to assess **indicators of the care continuum** appropriate? The statistical techniques must be appropriate to the data. |  |  |  |
| 1. Have appropriate confidence intervals been calculated and reported? |  |  |  |
| For the following questions, answer whether or not the following indicators were presented as part of the care continuum. |  |  |  |
| 1. HIV TESTING: Was it included? |  |  |  |
| 1. DIAGNOSED: Was it included |  |  |  |
| 1. ON ART TREATMENT: Was it included? |  |  |  |
| 1. VIRALLY SUPPRESSED: Was it included? |  |  |  |

1. Are the numerators for all indicators on the care cascade along with the total number among who each of the indicators were assessed clearly reported? If only some reported, the answer would be “no.”

2. Some criteria for determining if care continuum indicators were “clearly defined” include:

HIV testing: Reports how testing was assessed (e.g. self-reported, record review) and the timeframe (e.g. ever, in the past 12 months?)

Diagnosed: Reports how diagnosis was assessed (e.g. self-report, record review)On antiretroviral therapy (ART): Reports how ART status was assessed (e.g. self-report, record review, biological assessment)

Virally suppressed: Reports how viral suppression was assessed (e.g. self-report, record review, biological assessment) . For the viral suppression endpoint, it is expected that the study would have assessed RNA viral load or confirmed viral suppression status through a record review.

In order for this question to receive an answer of “yes,” the care continuum indictors must be clearly defined and they must have been assessed consistently across all participants.

3. In order for this question to receive an answer of “yes,” indicators of the care continuum must have been assessed consistently across all study participants. For example, if data on HIV testing is missing for a number of participants and this missingness was not accounted for, the answer would be “no.”

4. Were sampling weights included if appropriate? For time-location sampling and other venue-based sampling, were sampling weights according to venue considered? For respondent-driven sampling studies, were RDS adjustments included? For subsamples of individuals, RDS adjustments were not common before 2016, so this would still receive an answer of “yes” if no RDS adjustments were conducted. Was missingness adequately accounted for?

5. Some measure of uncertainty should be provided. For a “yes” response, appropriate confidence or credible intervals or error bars have been calculated around the estimate. Appropriate confidence or credible intervals have been calculated around the estimate.

6 through 9. These questions will receive an answer of “yes” if the indicator is present *as part* of the care continuum.

**Population Size Estimates (total pts: 5)**

| Criteria | **Yes** | **No** | **Add. paper utilized (Specify):** |
| --- | --- | --- | --- |
| BRIEFLY STATE THE OBJECTIVE OF THE SIZE ESTIMATION EXERCISE: |  |  |  |
| 1. Was the study population clearly specified and defined? |  |  |  |
| 1. Will the study population defined answer the research question proposed (adequately represent the target population)? |  |  |  |
| 1. Were methods of size estimation used reported? |  |  |  |
| 1. Were multiple methods used? |  |  |  |
| 1. Were the methods used to assess **population size** appropriate? |  |  |  |
| 1. Was some form of uncertainty presented, either a confidence interval or a range? |  |  |  |
| 1. Were implementation details sufficiently described? |  |  |  |

*1.* Did the authors describe their goal in conducting this research? Is it easy to understand what they were looking to find?

2. Did the authors describe the group of people from which the study participants were selected or recruited, using demographics, location, and time period? If you were to conduct this study again, would you know who to recruit, from where, and from what time period?

3.Some examples of methods of size estimation include service multiplier, unique object multiplier, capture-recapture, network scale-up, wisdom of the crowds, Delphi, program estimate, general population survey, mapping and enumeration, reverse tracking method, and literature review. Useful resource that describes the methods in greater detail: (<http://applications.emro.who.int/dsaf/EMROPub_2016_EN_19361.pdf>)

4. When multiple methods of size estimation are used to validate the direct estimate, this is generally considered to improve the quality of the estimate. Some methods used in conjunction include different multiplier methods as part of a larger population-based survey or an average of different program estimates of population size.

5. Were sampling weights included if appropriate? For time-location sampling and other venue-based sampling, were sampling weights by venue considered? For respondent-driven sampling studies, were RDS adjustments included? Was there any age-standardization or validation of estimates done?

6. Some measure of uncertainty should be provided. For a “yes” response, appropriate confidence or credible intervals or a range of estimates have been calculated. Appropriate confidence or credible intervals have been calculated around the estimate.

7. Some important implementation details include 1) in which specific geographic area size estimates were conducted (essential) and 2) a reproducible description of the method that was carried out. For example, in the case of a service multiplier, what service or program list was used to generate the estimate? For cap-recap, some details might include the number of venues attended during the first and second captures, the number of participants who were recaptured (overlap), etc. If no specific area is clearly presented, the answer would be “no.”
